# Green Extraction Techniques for Recovering Polyphenols From Cocoa Bean Shells for Application in Food Processing: A Systematic Review

**DOI:** 10.1111/1750-3841.71433

**Published:** 2026-09-13

**Authors:** Joy Chinenye Mba, Leda Atílio Pita, Helen Obioma Agu, Chioke Amaefuna Okolo, Carmen Sílvia Fávaro‐Trindade

**Affiliations:** ^1^ Faculdade de Zootecnia e Engenharia de Alimentos (FZEA) Universidade de São Paulo (USP) Pirassununga, São Paulo Brazil; ^2^ Department of Food Science and Technology Nnamdi Azikiwe University Awka Anambra Nigeria

**Keywords:** antioxidant activity, cocoa bean shells, food processing applications, green extraction techniques, polyphenols, sustainable extraction, waste valorization

## Abstract

Cocoa bean shells (CBSs), a by‐product of cocoa processing, are an underutilized source of bioactive polyphenols with strong potential for functional food applications. Although several studies and reviews have investigated polyphenol extraction techniques, gaps remain in consolidating findings, optimizing extraction conditions, and understanding the synergistic effects of combined green extraction methods under comparable conditions. This systematic review critically evaluates environmentally sustainable approaches for maximizing polyphenol recovery from CBS for food applications. Literature searches were conducted in Web of Science, Scopus, and PubMed, and studies were selected according to the PICOS (population, intervention, comparison, outcomes, and study design) framework and PRISMA (Preferred Reporting Items for Systematic Reviews and Meta‐Analyses) guidelines. Thirty‐six articles published between 2010 and 2025 across 15 countries were included. The findings showed that green extraction technologies generally demonstrated higher polyphenol recovery, enhanced the stability and antioxidant activity of the recovered polyphenols, reduced extraction time, and lowered solvent consumption under optimized extraction conditions. However, combining green extraction techniques did not significantly increase total phenolic content compared with single‐technique approaches, suggesting that process intensification does not always improve extraction efficiency. Considerable variability in reported total phenolic content was observed in the reviewed papers due to differences in extraction conditions, solvent systems, and reporting units. Overall, CBS demonstrated strong potential as a sustainable source of polyphenols, while green extraction technologies provide a promising strategy for valorizing CBS into high‐value ingredients for functional foods, nutraceuticals, and natural preservatives.

**Abbreviations**
CCD: central composite design
CBH: cocoa bean husk
CBS: cocoa bean shells
CH: cocoa hulls
DES: deep eutectic solvent
DM: dry matter
EAE: enzyme‐assisted extraction
HVED: high‐voltage electric discharge
MAE: microwave‐assisted extraction
NADES: natural deep eutectic solvents
PICOS: population, intervention, comparison, outcomes, and study design
PRISMA: Preferred Reporting Items for Systematic Reviews and Meta‐Analyses
PLE: pressurized liquid extraction
PEF: pulsed electric field
RSM: response surface methodology
SFE: supercritical fluid extraction
SWE: subcritical water extraction
SLR: systematic literature review
TPC: total phenolic content
UAE: ultrasound‐assisted extraction

## 1 Introduction

Large quantities of by‐products are generated during cocoa processing. Just 10% of the entire weight of cocoa fruit is used for commercial reasons, with the remaining 90% being thrown away as waste or by‐products, according to Rojo‐Poveda et al. (2020) and C. Soares, Moreira, et al. (2023). Cocoa bean shells (CBSs), the outermost cuticle covering cocoa beans, are one of several by‐products that are generated during the roasting process. Approximately 10%–17% of the weight of all cocoa beans is composed of CBS, and research has shown that these percentages may vary with processing conditions (Zhao and Fleet 2014; Hashimoto et al. 2018).  West Africa produces about 70% of the world's supply of cocoa. Ivory Coast is the largest producer (36%), followed by Indonesia and Ghana (World Population Review 2024). Brazil ranks sixth, with approximately 200,000 tons per year (Instituto Arapyaú 2024). Based on the estimated proportion of CBS generated during cocoa processing, Brazil may generate approximately 20,000–34,000 tons of CBS annually. This by‐product will accumulate due to the growing demand for cocoa beans, posing a significant disposal challenge that may worsen if appropriate waste‐management regulations are not implemented (Barišić et al. 2020).

Interestingly, CBS is rich in bioactive compounds, particularly polyphenols, which offer various health benefits, including antioxidant and anti‐inflammatory properties (Cooper et al. 2008; Younes et al. 2023). It contains bioactive peptide fractions (vicilin and albumin) with anti‐obesogenic and anti‐diabetic properties (Domínguez‐Pérez et al. 2020), as well as phytosterols (stigmasterol and campesterol), which possess anti‐inflammatory, anti‐tumoral, antibacterial, and antifungal properties (Younes et al. 2023). Notably, polyphenol‐rich extracts from CBS have demonstrated applicability as natural antioxidants to inhibit lipid oxidation in food products and as functional ingredients to enhance nutritional profiles (Ismail and Yee 2006; Manzano et al. 2017; Hernández‐Hernández et al. 2019). These reasons have led to a surge in interest in using this by‐product as a source of bioactive compounds in recent years, aligning with the increasing demand for natural, clean‐label ingredients and additives, as well as sustainable food‐processing practices (Okiyama et al. 2017; Rojo‐Poveda et al. 2020).

Extraction techniques have been used to separate bioactive compounds, such as polyphenols, from their biological sources. Efficient extraction methods for recovering polyphenols remain a critical focus, as they can promote sustainability by valorizing agricultural waste and reducing environmental impact (C. Soares, Moreira, et al. 2023; Fredsgaard et al. 2026).​ Conventional extraction techniques, such as Soxhlet extraction and maceration, are still widely utilized (Ueda et al. 2022; Osorio‐Tobón 2020), although they typically rely on organic solvents such as methanol, chloroform, or acetone, which pose environmental and health concerns due to their toxicity and low biodegradability. Major drawbacks of these techniques include the need for large volumes of extraction solvents, extended extraction times, high energy inputs, and the use of toxic solvents such as methanol and chloroform, which are limited in their application to food and pharmaceutical products (Quitério et al. 2022; C. Soares, Moreira, et al. 2023). To address these challenges, green extraction techniques have emerged as cost‐effective, innovative, and sustainable alternatives.

Several recent reviews have explored various green extraction strategies for recovering polyphenols from CBS, including ultrasound‐assisted extraction (UAE), microwave‐assisted extraction (MAE), pressurized liquid extraction (PLE), enzyme‐assisted extraction (EAE), deep eutectic solvent (DES) extraction, and supercritical fluid extraction (SFE), amongst others, and reported that these methods offer cost‐effective solutions for producing high‐quality extracts by improving extraction efficiency through disruption of the cell matrix, promoting mass transfer, and reducing temperatures, thereby preserving the antioxidant activity, compound stability and bioactivity of phenolic compounds, while minimizing degradation and contamination during processing (Ueda et al. 2022; Quitério et al. 2022; C. Soares, Moreira, et al. 2023; Quiceno‐Suarez et al. 2025). Other studies have examined the influence of process parameters, such as solvent composition, temperature, time, and solid–liquid ratio, on extraction efficiency, and polyphenol yield from CBSs (Quiceno‐Suarez et al. 2025; Jokić et al. 2018; Ruesgas‐Ramón et al. 2020; Mellinas et al. 2020).

Narrative reviews and studies examining recent advances in sustainable extraction approaches emphasize the growing shift toward environmentally friendly, low‐solvent, and energy‐efficient technologies (Fredsgaard et al. 2026; Hndeya et al. 2026). However, while these reviews provide valuable insights, they often adopt a broad or descriptive approach, lack critical comparisons of methodologies, and offer limited synthesis of quantitative outcomes across studies, leaving gaps in the evaluation of extraction efficiency, scalability, and suitability for food‐processing applications. Hence, this systematic review critically examines recent green extraction technologies applied to CBS for polyphenol recovery, focusing on comparisons of extraction performance, process optimization, and their potential integration into sustainable food processing systems. The rest of this paper is organized as follows: Section 2 covers the systematic literature review (SLR) method, including the search strategy and the PRISMA flow chart. Section 3 highlights the study's key findings. Section 4 discusses the results. Section 5 addresses the gaps in the literature and the study's limitations. Section 6 concludes this review by outlining future research directions and potential strategies to improve the efficiency, sustainability, and industrial applicability of green extraction technologies for the recovery of polyphenols from CBS. In addition to advances in extraction technologies, the safe use of CBS ‐derived ingredients requires compliance with regulatory requirements, contaminant control, and appropriate quality standards to facilitate their application in food systems.

Throughout this review, the term CBS refers specifically to the thin protective Testa that surrounds cocoa beans and is separated during roasting and winnowing. In the literature, this material is also referred to as *cocoa hulls* or *cocoa bean hulls*. In contrast, the term *cocoa husk* generally refers to the outer pod husk (pod shell), a distinct by‐product produced during pod opening. Because some published studies use these terms inconsistently, the original terminology employed by individual authors is acknowledged when describing specific studies; however, for consistency, the term CBS is used throughout this review except when directly referring to the terminology adopted in the cited publications.

## 2 Materials and Methods

### 2.1 Literature Search

A SLR was conducted to identify studies on green extraction techniques for recovering polyphenols from CBSs and their applications in food processing. The search covered three electronic databases: Scopus, Web of Science, and PubMed. The search was limited to studies published between 2010 and 2025, reflecting the growing interest in sustainable extraction technologies and in the valorization of agro‐industrial waste. Only articles published in English were considered in this review. Additional studies were identified through manual screening of reference lists of relevant review articles and primary research papers.

### 2.2 Search Strategy

The search strategy was developed based on four key concepts relevant to the objectives of this systematic review: CBSs, polyphenols, green extraction techniques, and food processing applications. Controlled vocabulary (e.g., MeSH terms, where applicable) and free‐text keywords related to these concepts were combined using Boolean operators (AND and OR) to maximize search sensitivity while maintaining specificity across Scopus, Web of Science, and PubMed.

#### 2.2.1 Study Selection and Boolean Operators

The Boolean logic used in the database search combined synonyms and related terms to maximize retrieval sensitivity while maintaining specificity. Search strings were adapted to meet the syntax requirements of each database. The search terms used based on the review objectives, along with summaries of search results across the databases, are presented in Tables 1 and 2, respectively. For the first objective (Identify green extraction techniques for CBS polyphenols), the following keywords were used (“cocoa bean shell” OR “cocoa shell” OR “cocoa husk” OR “cacao shell” OR “cocoa by‐product” OR “cacao by‐product”) AND (“green extraction” OR “sustainable extraction” OR “ultrasound” OR “ultrasound‐assisted” OR “microwave” OR “microwave‐assisted extraction” OR “enzyme‐assisted” OR “deep eutectic solvent” OR “DES” OR “NADES” OR “natural deep eutectic” OR “supercritical fluid” OR “SFE” OR “pressurized liquid extraction” OR “PLE”). This returned a total of 249 articles (PubMed = 22, Web of Science = 157, and Scopus = 70). For the second objective, which was to evaluate the polyphenol extraction efficiency and yield, used the following keywords (“polyphenol” OR “phenolic compounds” OR “flavonoids” OR “procyanidin” OR “catechin” OR “epicatechin”) AND (“yield” OR “extraction efficiency” OR “antioxidant activity”) and returned a total of 241,393 articles (PubMed = 22,549, Web of Science = 104,860, and Scopus = 113,984). The third objective was to assess polyphenol applications in food processing used the following keywords (“cocoa bean shell” OR “cocoa shell” OR “cocoa husk” OR “cacao shell” OR “cocoa by‐product” OR “cacao by‐product”) AND (“food” OR “beverage” OR “food application” OR “nutraceutical” OR “edible film” OR “packaging”), and returned a total of 936 articles (PubMed = 116, Web of Science = 498, and Scopus = 322) covering CBS polyphenols or CBS extraction studies worldwide, across three databases.

**TABLE 1:** Search terms based on review objectives.

Objective	Keywords	Search terms (Boolean strings)
Identify green extraction techniques for CBS polyphenols	Cocoa bean shells, green extraction	(“cocoa bean shell” OR “cocoa shell” OR “cocoa husk” OR “cacao shell” OR “cocoa by‐product” OR “cacao by‐product”) AND (“green extraction” OR “sustainable extraction” OR “ultrasound” OR “ultrasound‐assisted” OR “microwave” OR “microwave‐assisted extraction” OR “enzyme‐assisted” OR “deep eutectic solvent” OR “DES” OR “NADES” OR “natural deep eutectic” OR “supercritical fluid” OR “SFE” OR “pressurized liquid extraction” OR “PLE”)
Evaluate extraction efficiency and yield	Polyphenols, yield, antioxidant activity	(“polyphenol” OR “phenolic compounds” OR “flavonoids” OR “procyanidin” OR “catechin” OR “epicatechin”) AND (“yield” OR “extraction efficiency” OR “antioxidant activity”)
Assess applications in food processing	Food applications, functional foods	(“cocoa bean shell” OR “cocoa shell” OR “cocoa husk” OR “cacao shell” OR “cocoa by‐product” OR “cacao by‐product”) AND (“food” OR “beverage” OR “food application” OR “nutraceutical” OR “edible film” OR “packaging”)

**TABLE 2:** Summary of search results across databases with filters.

Databases	Initial search results			Final results with filters (publication year and English)		
	Objective 1	Objective 2	Objective 3	Objective 1	Objective 2	Objective 3
Scopus	70	113,984	322	70	92,360	259
Web of Science	157	104,860	498	147	88,002	413
PubMed	22	22,549	116	21	19,243	107
Total articles	249	241,393	936	238	199,605	779

**FIGURE 1:**
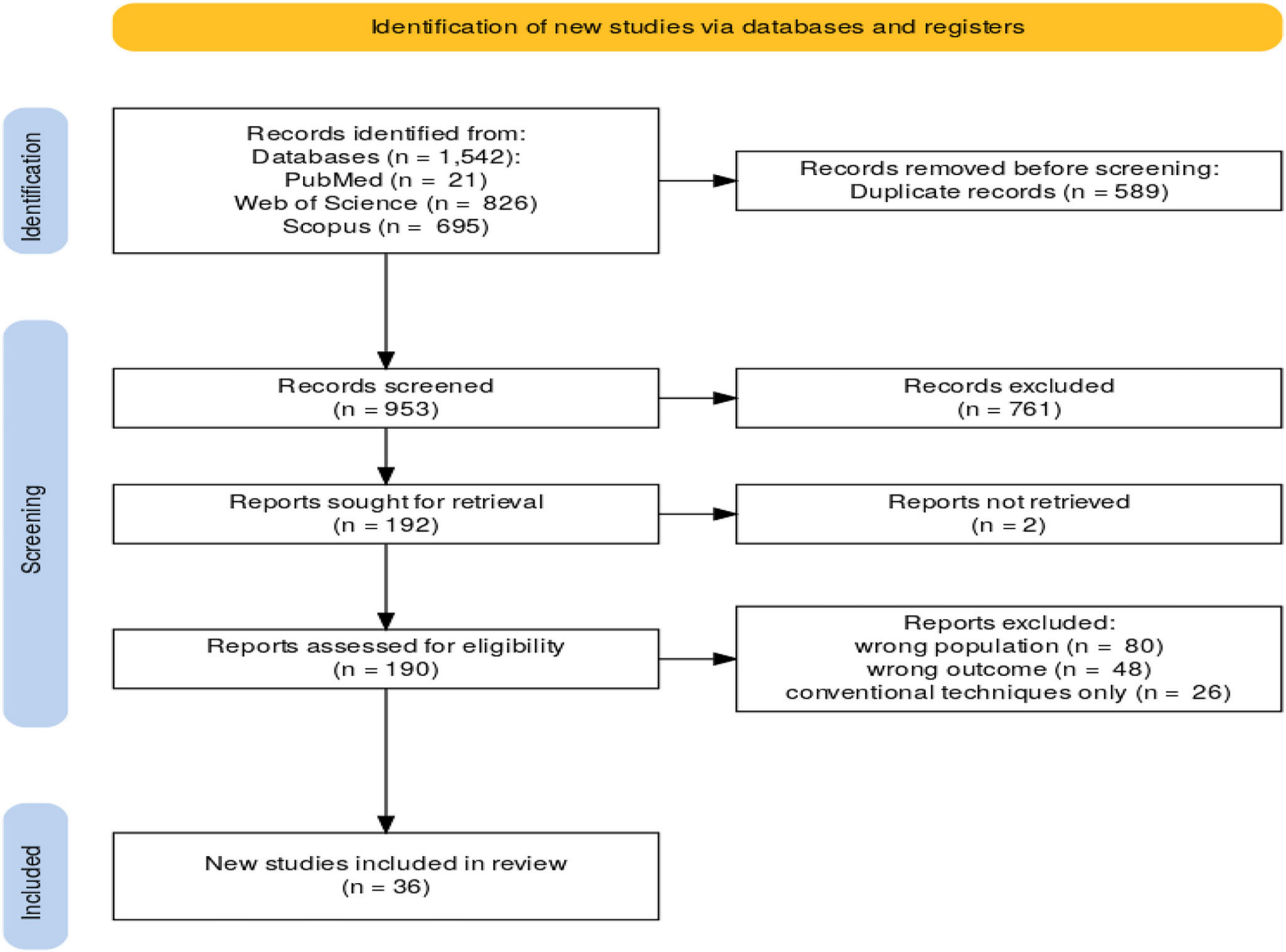
PRISMA flow diagram of the search and selection of studies included in the review.

The combined database searches initially retrieved 242,578 records across the three review objectives. After restricting the search to publications published between 2010 and 2025 and written in English, 200,622 records remained (Table 2). However, because the broad search strategy for Objective 2 was designed to maximize sensitivity, a substantial number of retrieved records were clearly unrelated to CBS or cocoa‐derived materials. Therefore, before exporting the records to Rayyan, a manual title‐based relevance screening was performed within each database. During this preliminary screening, only records whose titles indicated a clear focus on CBS, cocoa by‐products, cocoa husk, or other cocoa‐derived materials in relation to polyphenols, green extraction technologies, antioxidant properties, or food applications were retained for further evaluation. Records clearly unrelated to cocoa‐derived materials were excluded at this stage. Following this relevance screening, 1542 records were exported to Rayyan for duplicate removal and subsequent title and abstract screening. This procedure reduced the number of clearly irrelevant records while preserving studies potentially meeting the eligibility criteria, and improved the specificity of the review while maintaining a comprehensive and reproducible search strategy. The number of articles remaining for final analysis is shown in Figure 1.

This review included only studies published in English to ensure consistent interpretation of the available literature, as English is the principal language of scientific communication. Nevertheless, this criterion may have introduced language bias by excluding relevant studies published in other languages. Given that cocoa production and research are concentrated in regions where Spanish, Portuguese, and French are widely spoken, potentially valuable evidence may have been overlooked, thereby affecting the completeness of the synthesized findings.

### 2.3 Study Eligibility Criteria

This systematic review followed the Preferred Reporting Items for Systematic Reviews and Meta‐Analyses (PRISMA) guidelines. The PICOS (population, intervention, comparison, outcomes, and study design) model was used to develop the eligibility criteria. The Population was CBSs (*Theobroma cacao* L.) derived from any cocoa variety and origin. Interventions were Green or sustainable extraction methods, including, but not limited to, UAE, MAE, EAE, SFE, PLE, and DES‐based extraction. The comparator was conventional solvent extraction (maceration, Soxhlet) when reported.

#### 2.3.1 Inclusion and Exclusion Criteria

##### 2.3.1.1 Inclusion Criteria

- Studies investigating CBS as the primary raw material;
- Studies focusing on polyphenol extraction or characterization;
- Use of green or sustainable extraction techniques (e.g., UAE, MAE, SFE, PLE);
- Studies reporting quantitative data (e.g., yield, TPC, antioxidant activity);
- Articles discussing food or nutraceutical applications;
- Peer‐reviewed journal articles.

##### 2.3.1.2 Exclusion Criteria

- Studies not involving the CBS;
- Use of conventional extraction methods only;
- Review papers, book chapters, conference abstracts (unless used for background), editorials, patents, and theses without primary experimental data;
- Publications in languages other than English;
- Studies lacking sufficient methodological or quantitative data on polyphenol yield, composition, or bioactivity.

### 2.4 Study Selection

The study selection process followed the PRISMA guidelines. After the search, 1542 articles aligning with the objectives were downloaded from the electronic databases (Scopus, PubMed, and Web of Science) and exported to Rayyan (http://www.rayyan.ai) for screening and duplicate removal. A total of 1542 records were imported into Rayyan from the three electronic databases. Duplicate detection identified 1066 potential duplicate records, which were reviewed manually to ensure accuracy. Following manual verification, 589 records were confirmed as true duplicates and removed, leaving 953 unique records for title and abstract screening. The titles and abstracts of the remaining 953 studies were screened against the inclusion and exclusion criteria. Of the 953 screened articles, 710 studies were excluded because they did not focus on CBS. An additional 41 studies were excluded as the wrong publication type (reviews), 2 articles were excluded because they were not written in English, and 8 studies were not peer‐reviewed and were removed. A total of 192 articles were deemed eligible for full‐text reading and final evaluation. Note that 62 relevant articles met the inclusion criteria; 26 were excluded because they focused solely on conventional techniques. Thus, only 36 articles were included in this systematic review.

The process of selecting articles for analysis is illustrated in the PRISMA flow chart (Figure 1). Following duplicate removal, the remaining 953 articles underwent title, keyword, and abstract screening. To improve consistency and reduce selection bias, the articles were independently screened by pairs of reviewers using the predefined inclusion and exclusion criteria. Each reviewer classified studies as “include,” “exclude,” or “maybe” within the Rayyan platform. Following the initial screening stage, agreement between reviewers was assessed by comparing the classification decisions for each article. Articles receiving the same classification from both reviewers were automatically accepted or excluded accordingly. Articles with conflicting decisions (e.g., one reviewer selecting “include” and the other selecting “exclude” or “maybe”) were flagged as disagreements. These disagreements were identified and reviewed during a consensus meeting involving representatives from both reviewer groups.

For each disputed article, reviewers re‐examined the title, abstract, and, where necessary, the full text against the predefined eligibility criteria. If consensus could not be reached after discussion, a senior reviewer (C.S.F.T.) independently evaluated the study and made the final determination regarding eligibility. Following the resolution of all disagreements, J.C.M. compiled the preliminary list of eligible studies and circulated it to L.A.P., C.A.O., and H.O.A. for an additional quality review. A final meeting was then conducted to verify study eligibility, ensure consistent application of the selection criteria, and confirm the final set of studies included in the review. All inclusion and exclusion decisions were therefore based on reviewer consensus or adjudication by an independent reviewer when consensus could not be achieved.

### 2.5 Data Extraction and Synthesis

Four reviewers used a standardized data extraction form to obtain and report relevant information, such as country of publication, authors, publication year, pretreatment given to CBS prior to polyphenol extraction,  extraction method, extraction solvent and conditions (time, temperature, energy input), polyphenol yield and composition (mg GAE/g CBS or equivalent), antioxidant assays and bioactivity results (DPPH, ABTS, FRAP, ORAC etc.), food application mentioned, and key findings from the study. If data or information were missing or unclear, it was assumed that the authors did not report these variables. For this systematic review, data from eligible studies are presented in Table 3, and a narrative synthesis of the results is provided, based on the main characteristics of the reviewed articles and findings.

## 3 Results

### 3.1 Study Selection

The PRISMA guidelines were followed to develop a flow diagram illustrating the literature search and study selection process (Figure 1). The initial database search identified 1542 articles (PubMed: 21; Web of Science: 826; Scopus: 695). After the removal of duplicates (*n* = 589), 953 articles remained for abstract screening. Following this stage, 192 articles were considered eligible for full‐text review and were retrieved for further assessment. Ultimately, 36 studies met all the inclusion criteria after full‐text evaluation and were selected for data extraction and inclusion in this systematic review.

### 3.2 Geographical Description of the Study

**FIGURE 2:**
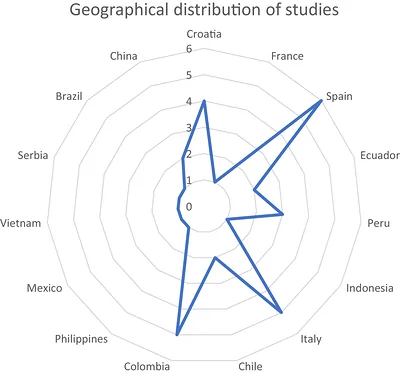
Radar plot of the geographical distribution of studies.

The reviewed literature has increased considerably in recent years, with half of the included studies published between 2023 and 2024. Geographically, research activity was distributed across fifteen countries, with Spain contributing the largest number of studies, followed by Italy and Colombia (Figure 2). The studies are distributed as follows: Spain (*n* = 6; Mellinas et al. 2020; Rebollo‐Hernanz et al. 2021; Sánchez et al. 2023; Gil‐Ramírez et al. 2024; Sánchez, Bernal, Laca et al. 2024, Sánchez, Penin, Laca et al. 2024). Italy (*n* = 5; Disca et al. 2024; Grassia et al. 2021; Barbosa‐Pereira et al. 2018; Papillo et al. 2019; Grillo et al. 2019), Colombia (*n* = 5; Quiceno‐Suarez et al. 2025; Garzón‐Serrano et al. 2025; Quiceno‐Suárez et al. 2024; Ramos et al. 2023; Pico Hernández et al. 2019). Other countries were: Croatia (*n* = 4; Jokić et al. 2020; Barišic et al. 2020; Jokić et al. 2018; Jokić et al. 2019), Indonesia (*n* = 1; Rahayu et al. 2019), France (*n* = 1; Ruesgas‐Ramón et al. 2019), Ecuador (*n* = 2; Avilés et al. 2019; Llerena et al. 2023), Peru (*n* = 3; Valencia et al. 2024; Ramos‐Escudero et al. 2023; Ramos‐Escudero et al. 2024), Brazil (*n* = 1; I. D. Soares, Cirilo, et al. 2023), China (*n* = 2; Zhong et al. 2019; Nsor‐Atindana et al. 2012), Chile (*n* = 2; Benítez‐Correa et al. 2023; Benítez‐Correa et al. 2024), Philippines (*n* = 1; Duque et al. 2024), Mexico (*n* = 1; Velasquez‐Jimenez et al. 2023), Vietnam (*n* = 1; Huynh et al. 2023), and Serbia (*n* = 1; Švarc‐Gajić et al. 2023).

### 3.3 Study Description

The source of polyphenol in all articles reviewed was CBS or cocoa bean husks (CBH), or cocoa hulls (CH), obtained as a by‐product from the processing of cocoa beans (*T. cacao*) into chocolate, beverage, or other food uses. CBS was subjected to various pretreatments prior to polyphenol extraction. The pretreatments included washing, fermentation, enzymatic treatment, defatting, drying, roasting, grinding, and sieving to obtain uniformly sized particles. The extraction solvents utilized included ethanol, water, methanol, acetone, glacial acetic acid solutions, hydroalcoholic solutions, DES, and n‐heptane. Extraction techniques included conventional ethanol extraction, maceration, SFE, pulsed electric field (PEF), MAE, UAE, DES extraction, ohmic heating (OH), hydrothermal extraction (HTE) (autohydrolysis), and high‐voltage electric discharge (HVED) extraction. Total phenolic content (TPC) and polyphenol extraction yields were determined qualitatively and quantitatively using the Folin‐Ciocalteu and HPLC techniques, while antioxidant capacities were reported as IC50 or EC50 values using DPPH, ABTS, ORAC, TEAC, and FRAP methods. Most of the articles reported practical applications in food products, including cocoa beverages, tisanes, black tea, capsules, tea bag beverages, high‐fiber functional biscuits, cookies, chocolate bars, iced tea, herbal infusions, dairy desserts, and extruded snack products.

### 3.4 Key Findings From Studies

A summary of the findings, including extraction technique, typical extraction conditions, major bioactive compound recovered, reported extraction outcomes, and key findings from the reviewed papers, is presented in Table 3.   (+)‐Catechin, epicatechin, and procyanidins B1 and B2 were found to be the main polyphenols in CBS (Jokić et al. 2018; Jokić et al. 2019). Factors such as genotype variation, ripeness, fermentation, climate, soil conditions, and stress can influence the polyphenol content in CBS (Mellinas et al. 2020; Sánchez et al. 2023). While pretreatment conditions like light roasting and autohydrolysis were reported to increase the polyphenol content (Sánchez, Bernal, Laca et al. 2024), prolonged fermentation, intense roasting temperatures, and longer enzymatic treatments decreased the polyphenol and antioxidant activities (Ramos‐Escudero et al. 2024; Huynh et al. 2023; Sánchez, Bernal, Laca et al. 2024; Disca et al. 2024). Reduction of CBS particle size, either through grinding or milling, increased extraction yields and polyphenol content (Pico Hernández et al. 2019; Grassia et al. 2021). PEF pretreatment improved polyphenol extraction relative to conventional extraction in 75% of CBS samples (Barbosa‐Pereira et al. 2018).

**TABLE 3:** Summary of studies investigating green extraction technologies for recovery of phenolic compounds from cocoa bean shells.

Extraction technique	Typical extraction conditions	Major bioactive compounds recovered	Reported extraction outcomes	Key findings	Representative studies
Ultrasound‐assisted extraction (UAE)	40–70% ethanol; ultrasonic bath/probe; 20–60 min	Phenolic acids, flavonoids, methylxanthines	Higher TPC and antioxidant capacity than CSE	Cavitation enhances cell disruption and mass transfer while reducing extraction time.	Ramos et al. (2023), Valencia et al. (2024), Llerena et al. (2023), Quiceno‐Suarez et al. (2025), Gil‐Ramírez et al. (2024), Grassia et al. (2021), Avilés et al. (2019), Grillo et al. (2019), Duque et al. (2024)
Microwave‐assisted extraction (MAE)	Microwave heating with optimized pH, temperature, and time	Theobromine, epicatechin, caffeic acid, chlorogenic acid, ferulic acid	High extraction yield with short processing time	Process optimization significantly improves recovery while reducing extraction time compared with conventional extraction.	Mellinas et al. (2020), Nsor‐Atindana et al. (2012), Rahayu et al. (2019)
Subcritical water extraction (SWE)	Water (120°C–220°C) under pressure	Total phenolics, caffeine, theobromine	High TPC and antioxidant activity at elevated temperatures	Temperature is the principal factor controlling extraction efficiency.	Jokić et al. (2018), Pico Hernández et al. (2019), Švarc‐Gajić et al. (2023)
High‐voltage electric discharge (HVED)	Water‐based extraction using optimized discharge frequency, treatment time, and solvent ratio	Catechin, epicatechin, gallic acid, caffeine, theobromine	Enhanced extraction compared with conventional water extraction under optimized conditions	Extraction efficiency depends strongly on electrical parameters; excessive treatment may reduce phenolic recovery.	Jokić et al. (2019), Barišić et al. (2020)
Pulsed electric field (PEF)	Short electrical pulses followed by solvent extraction	Polyphenols, flavonoids	Increased TPC compared with untreated samples	Cell electroporation enhances solvent penetration while preserving thermolabile compounds.	Barbosa‐Pereira et al. (2018), Benítez‐Correa et al. (2024)
Deep eutectic solvent (DES) extraction	Choline chloride‐based DES	Phenolics and flavonoids	High recovery using environmentally friendly solvents	DES improves extraction while reducing reliance on organic solvents.	Benítez‐Correa et al. (2023), Ruesgas‐Ramón et al. (2020)
Ohmic heating (OH)	Electrical heating with aqueous ethanol	Phenolics and flavonoids	Approximately 50% greater extraction efficiency than conventional heating	Rapid and uniform heating improves extraction efficiency while reducing thermal degradation.	Sánchez et al. (2023)
Hydrothermal extraction (autohydrolysis)	Pressurized hot water	Phenolics and flavonoids	Increased extraction yields up to 56.5% compared with conventional solvent extraction	Elevated temperatures reduce water polarity and viscosity while increasing diffusivity, enhancing the solubility and mass transfer of phenolic compounds	Sánchez, Bernal, Laca et al. 2024; Sánchez, Penin, Laca et al. 2024

Ethanol was the most commonly used green solvent for CBS polyphenol extraction, and its concentration determined the quantity of polyphenols extracted. The highest yield of polyphenols was obtained when concentrations of ethanol were less than 50%, while concentrations greater than 50% reduced the yield and content of polyphenols (Valencia et al. 2024; Ramos‐Escudero et al. 2024; Sánchez et al. 2023). Also, ethanol–water mixtures emerged as the most practical food‐grade solvent system across most studies, although several acetone‐ and methanol‐based mixtures achieved higher analytical recoveries (Belščak‐Cvitanović et al. 2018; Duque et al. 2024; Ramos‐Escudero et al. 2024). For instance, the acetone/water/acetic acid (70:29.5:0.5) mixture extracted CBS polyphenols more efficiently (Gil‐Ramírez et al. 2024). Some studies also reported that shorter extraction times were associated with lower polyphenol recovery (Rebollo‐Hernanz et al. 2021).

Across comparative studies, green extraction technologies—including UAE, SWE, MAE, HVED, DES, and PEF—consistently outperform conventional solvent‐based methods, such as maceration and Soxhlet extraction, in terms of extraction yield, polyphenol recovery, and antioxidant activity from CBS. These improvements are typically accompanied by reduced extraction times and lower solvent consumption, highlighting the efficiency advantages of process intensification strategies (Jokić et al. 2018; Ruesgas‐Ramón et al. 2019; Barišić et al. 2020; Mellinas et al. 2020; Jokić et al. 2020; Ramos et al. 2023; Švarc‐Gajić et al. 2023; Benítez‐Correa et al. 2023; Quiceno‐Suárez et al. 2024). Collectively, these findings confirm the suitability of green extraction approaches for the sustainable valorization of CBS.

However, combining multiple extraction technologies does not always result in additive or synergistic improvements in TPC. In some cases, hybrid systems such as ultrasound‐assisted microwave extraction (UAE‐MAE) show no significant advantage over optimized single‐technique processes, suggesting that once efficient cell disruption is achieved, further process intensification may yield diminishing returns (Disca et al. 2024). In contrast, integrating complementary pretreatments, such as EAE prior to SFE, can significantly enhance phenolic recovery in structurally resistant matrices by improving substrate accessibility (Quiceno‐Suarez et al. 2025). These contrasting outcomes indicate that the effectiveness of combined technologies depends strongly on matrix characteristics and the mechanistic compatibility of the processes involved.

In addition, the presence of complex food matrices can limit extraction efficiency by restricting solvent access and mass transfer, thereby reducing yields and phenolic recovery compared with simplified systems (Grassia et al. 2021; Valencia et al. 2024). This reinforces the need to consider matrix effects alongside process optimization when evaluating extraction performance and designing integrated green extraction strategies for CBS valorization.

### 3.5 Risk of Bias Assessment

**FIGURE 3:**
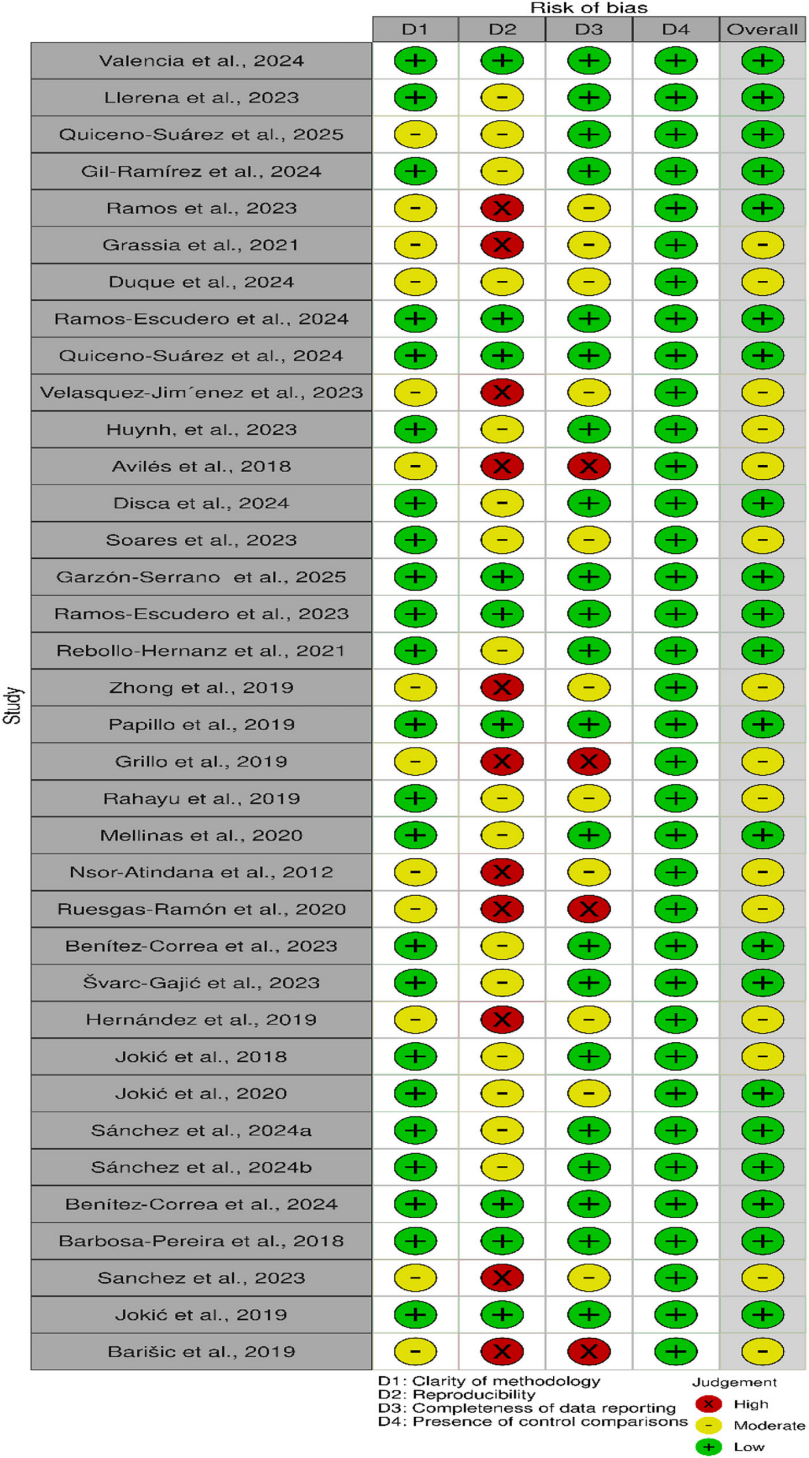
Risk of bias of reviewed papers. (× red) = high; (+ green) = low; (− yellow) = moderate.

A risk‐of‐bias assessment was conducted for all 36 included laboratory‐based extraction studies using predefined evaluation criteria adapted from methodological quality assessment frameworks relevant to experimental extraction research (Figure 3). Because no validated risk‐of‐bias instrument (such as RoB 2, ROBINS‐I, QUADAS‐2, and the Newcastle–Ottawa Scale, which are primarily intended for randomized clinical trials, non‐randomized intervention studies, diagnostic accuracy studies, or observational research) currently exists for laboratory‐based studies evaluating green extraction technologies, an adapted assessment framework was developed to evaluate methodological quality across four domains considered critical for extraction studies: (i) clarity of methodology, (ii) reproducibility of extraction conditions, (iii) completeness of data reporting, and (iv) presence of control or comparative methods.

Each study was independently evaluated and categorized as having low, moderate, or high risk of bias within each domain. Studies were classified as low risk when sufficient methodological detail and appropriate experimental procedures were reported, moderate risk when reporting was incomplete or insufficiently detailed, and high risk when major methodological limitations or substantial omissions were identified. The assessment criteria are presented in Table 4, while the RobVis tool was used exclusively to visualize and summarize the results.

**TABLE 4:** Criteria used for risk of bias assessment and classification of included studies.

Risk of bias domain	Low risk of bias	Moderate risk of bias	High risk of bias
Clarity of Methodology (D1)	Experimental procedures, materials, extraction methods, and analytical techniques are clearly and comprehensively described	The methodology is partially described, with some missing experimental details	The methodology is poorly described or insufficient for understanding the study design
Reproducibility of Extraction Conditions (D2)	Extraction conditions (e.g., solvent type, solvent ratio, temperature, time, pressure, particle size) are fully reported and reproducible	Some extraction parameters are reported, but important conditions are missing or unclear	Extraction conditions inadequately reported, preventing reproducibility
Completeness of Data Reporting (D3)	Key outcomes such as extraction yield, total phenolic content, antioxidant activity, and statistical data are fully reported	Some relevant results or statistical details are missing	Major outcomes, statistical analysis, or important data were not reported
Presence of Control Comparisons (D4)	Appropriate control or comparison treatments included (e.g., conventional extraction methods or optimized conditions)	Limited or insufficient comparison/control information provided	No control or comparison treatment was included
Overall Risk of Bias Classification	Studies with most domains rated as low risk and no major methodological concerns	Studies containing one or more unclear domains but without critical methodological weaknesses	Studies with multiple high‐risk domains or serious methodological limitations affecting reliability

It is important to acknowledge that these criteria were adapted rather than formally validated. Although the framework was designed to address methodological issues that directly influence the reliability and reproducibility of laboratory extraction studies, its application entails some reviewer judgment and has not been externally validated for this type of research. Consequently, the risk‐of‐bias assessment should be interpreted as a structured appraisal of methodological quality rather than a definitive or standardized measure of bias. This limitation should be considered when comparing methodological quality across studies.

The risk‐of‐bias assessment revealed that although most included studies demonstrated acceptable methodological quality, important limitations remain that may affect the reliability, reproducibility, and comparability of findings across green extraction technologies for recovering polyphenols from CBS. Overall, 58.3% of the included studies were classified as low risk of bias, while 41.7% were classified as having some methodological concerns. No study met the criteria for an overall high risk of bias, although several exhibited weaknesses in individual domains, particularly reproducibility. While these findings generally support the credibility of the available evidence, the substantial proportion of studies with methodological concerns, together with the use of an adapted assessment framework, indicates that comparisons among studies should be interpreted cautiously.

#### 3.5.1 Clarity of Methodology

Among the assessed domains, clarity of methodology (D1) demonstrated relatively strong performance, with 63.9% of studies classified as low risk of bias and none categorized as high risk. This suggests that most studies adequately described their extraction procedures, solvents, analytical techniques, and processing conditions. Adequate methodological reporting is particularly important in extraction studies because extraction efficiency is highly dependent on operational variables such as solvent polarity, extraction temperature, extraction time, and pretreatment conditions. Nevertheless, more than one‐third of the studies were still rated as having some concerns due to incomplete reporting of experimental details. In several cases, insufficient information on sample pretreatment, particle‐size distribution, or extraction optimization limited experimental reproducibility and hindered direct comparisons among studies.

#### 3.5.2 Reproducibility

Reproducibility of extraction conditions (D2) emerged as the weakest methodological domain, with only 25% of studies classified as low risk, 44.4% as having some concerns, and 30.6% as high risk. This finding represents a critical limitation in the literature studied. Green extraction technologies such as UAE, MAE, PLE, and SFE are highly sensitive to operational conditions. Small variations in extraction parameters can substantially alter polyphenol yield, antioxidant activity, selectivity, and compound stability. However, many studies failed to comprehensively report essential parameters, including solvent‐to‐solid ratio, extraction pressure, power intensity, moisture content, extraction cycles, and equipment specifications. As a result, reproducibility across laboratories becomes difficult, reducing confidence in the consistency and scalability of reported extraction efficiencies. The high proportion of studies with concerns in this domain also highlights the absence of standardized reporting guidelines for CBS polyphenol extraction studies.

#### 3.5.3 Completeness of Data Reporting

Completeness of data reporting (D3) showed moderate methodological quality, with 58.3% of studies classified as low risk, 30.6% as having some concerns, and 11.1% as high risk. Although most studies reported TPC and antioxidant activity, several lacked comprehensive statistical analysis, replication details, confidence intervals, or full characterization of the extracted compounds. In some cases, extraction yields were reported without sufficient description of the analytical methodology or the sample basis, complicating comparisons across studies. In addition, inconsistent use of reporting units (e.g., mg GAE/g, g GAE/100 g, mg/L) further reduced comparability among extraction techniques. Inadequate data reporting limits the ability to conduct quantitative comparisons and weakens the robustness of evidence synthesis in systematic reviews.

#### 3.5.4 Presence of Control or Comparative Methods

In contrast, the presence of control comparisons (D4) demonstrated the strongest methodological performance, with all studies classified as low risk of bias. This indicates that most studies included comparative extraction methods (conventional techniques), thereby strengthening the interpretation of extraction performance. The consistent inclusion of controls improves the internal validity of the studies and supports more reliable conclusions about the effectiveness of emerging green extraction technologies compared with conventional methods.

#### 3.5.5 Overall Risk of Bias

The overall risk‐of‐bias assessment indicated that the included studies on green extraction technologies for recovering polyphenols from CBSs demonstrate generally acceptable methodological quality, although several limitations affecting reproducibility and transparency in reporting remain evident. Of the included studies, 58.3% (21/36) were classified as having a low overall risk of bias, while the remaining 41.7% (15/36) were rated as having a moderate overall risk of bias. Importantly, no study was judged to have a high overall risk of bias, indicating that most studies employed reasonably sound experimental approaches and provided sufficient methodological detail to support the reliability of their findings.

Despite the generally favorable overall risk‐of‐bias profile, the findings suggest that methodological heterogeneity remains a significant challenge within the field. Variations in raw material characteristics, fermentation and roasting conditions, particle size, solvent systems, extraction parameters, analytical techniques, and reporting practices contribute to substantial inter‐study variability. Consequently, although the current evidence supports the potential of green extraction technologies to recover CBS polyphenols, direct comparisons of extraction efficiencies across studies should be interpreted with caution.

The assessment also suggests that recent studies tend to demonstrate improved methodological quality compared with earlier publications, likely reflecting growing awareness of reproducibility, sustainability, and standardization requirements in green extraction research. However, the persistence of methodological concerns emphasizes the need for harmonized experimental protocols, standardized reporting criteria, and more rigorous analytical validation in future studies. Improved consistency in reporting extraction parameters, statistical analyses, and polyphenol quantification methods would substantially strengthen the comparability, reproducibility, and industrial applicability of CBS polyphenol extraction research.

## 4 Discussion

### 4.1 Genetic Makeup and Pretreatment of CBS

The polyphenol content of CBS is determined by a complex interaction between genetic, environmental, and processing factors, resulting in considerable variability among studies. Genotype, fruit maturity, climatic conditions, soil characteristics, and environmental stress influence the accumulation of flavan‐3‐ols, procyanidins, and other phenolic compounds in cocoa tissues before processing (Mellinas et al. 2020; Sánchez et al. 2023; Rojo‐Poveda et al. 2020; Constante Catuto et al. 2024). Consequently, differences in polyphenol recovery reported across studies may reflect not only extraction efficiency but also variations in the intrinsic composition of the CBS raw material. This highlights the importance of considering genetic origin and growing conditions when comparing extraction outcomes.

Among postharvest factors, fermentation is a key determinant of polyphenol retention in CBSs. Across studies, extended fermentation is generally associated with reduced TPC and antioxidant activity, largely due to diffusion of phenolic compounds from storage tissues, enzymatic oxidation, polymerization reactions, and interactions with proteins and polysaccharides that limit phenolic availability (Disca et al. 2024; Benítez‐Correa et al. 2024; Llerena et al. 2023; Velásquez‐Jiménez et al. 2023). Similar degradation pathways have been widely reported in the literature, reinforcing the role of oxidation and condensation reactions as primary drivers of phenolic loss during fermentation Adebo and Gabriela Medina‐Meza 2020). These findings highlight fermentation as a critical link between flavor development and the preservation of bioactive compounds, with longer processing times potentially compromising phenolic integrity. This is further supported by evidence showing that shorter fermentation durations can retain higher phenolic levels, indicating that extraction yield and the preservation of bioactive compounds do not necessarily increase in parallel (Utami et al. 2016).

Thermal pretreatments exhibit a similarly complex influence on polyphenol recovery. Dry roasting is generally associated with decreases in TPC and antioxidant activity, particularly at higher temperatures, due to thermal degradation and oxidative reactions affecting phenolic compounds (Huynh et al. 2023; Disca et al. 2024; Agus et al. 2018; Gumelar and Mochamad 2022). However, the impact of heat is not uniformly negative across all thermal processes. In contrast to dry roasting, hydrothermal pretreatments can enhance phenolic extractability by disrupting the lignocellulosic matrix, increasing cell wall permeability, and facilitating the release of bound phenolics (Sánchez, Bernal, Laca et al. 2024; Huynh et al. 2023). These contrasting outcomes indicate that the effect of heat on polyphenol recovery is highly dependent on the processing environment: dry heat tends to promote degradation, whereas controlled aqueous thermal systems may improve the accessibility of bound compounds and increase extractable phenolic fractions.

Particle‐size reduction was another pretreatment factor that consistently improved polyphenol recovery across studies. Grinding and milling increase the surface area available for solvent interaction, enhance solvent penetration, and reduce mass‐transfer limitations during extraction (Pico Hernández et al. 2019; Botella‐Martínez et al. 2021; Grassia et al. 2021). Both Botella‐Martínez et al. (2021) and Grassia et al. (2021) reported higher concentrations of free and bound phenolics in finer CBS fractions, indicating that particle‐size reduction facilitates the release of intracellular phenolic compounds. Nevertheless, the available evidence also suggests that excessive particle‐size reduction may increase processing costs and create operational challenges during industrial‐scale extraction. Therefore, particle size optimization should balance improvements in extraction efficiency with practical processing considerations.

Generally, the reviewed literature demonstrates that pretreatment conditions can influence CBS polyphenol recovery as strongly as the extraction technology itself. Fermentation and severe roasting generally reduce phenolic retention, whereas hydrothermal processing and particle‐size reduction enhance phenolic extractability. These findings emphasize that maximizing polyphenol recovery from CBS requires an integrated approach in which raw material selection, postharvest processing, pretreatment, and extraction conditions are optimized simultaneously rather than considered independently.

### 4.2 Extraction Solvent and Conditions

The efficiency of polyphenol extraction from CBS is governed primarily by solvent polarity and extraction conditions, with the reviewed studies consistently demonstrating that extraction performance depends on the interaction between these factors rather than on any single parameter. Despite the diversity of extraction technologies investigated, a clear consensus emerges that solvent composition is one of the most important determinants of TPC, antioxidant activity, and overall extraction yield.

Across the reviewed studies, aqueous organic solvent systems consistently outperform pure solvents in extracting phenolic compounds from CBSs. Ethanol–water mixtures, particularly those containing intermediate ethanol concentrations, generally achieve greater polyphenol recovery than either water alone or highly concentrated ethanol solutions (Valencia et al. 2024; Ramos‐Escudero et al. 2024; Barbosa‐Pereira et al. 2021). This behavior reflects the structural diversity of CBS phenolics, which encompass compounds with a wide range of polarities. Intermediate ethanol concentrations provide an optimal balance of solvent polarity, enhancing both the solubilization of structurally diverse phenolics and solvent penetration into the lignocellulosic matrix (Kaczorová et al. 2021). The consistency of this trend across different extraction technologies and experimental conditions suggests that solvent composition is a primary determinant of extraction efficiency, reinforcing the importance of optimizing solvent polarity to maximize phenolic recovery.

Although ethanol is the most widely used green solvent, the reviewed literature indicates that solvent mixtures with additional organic components can further enhance extraction efficiency. Acetone–water–acetic acid mixtures (Garzón‐Serrano et al. 2025), methanol–water–acetone systems (Araújo De Barros et al. 2023), and aqueous acetone extracts (Nsor‐Atindana et al. 2012) frequently yielded higher TPC values than ethanol alone. Together, these findings suggest that maximizing phenolic recovery often requires tailoring solvent polarity to the target phenolic profile rather than relying on a single universal solvent system. However, because acetone and methanol present environmental and toxicological concerns, their practical application in food and nutraceutical industries may be more limited than that of ethanol–water systems.

Beyond solvent composition, extraction conditions consistently influenced polyphenol recovery. Extraction time, temperature, solid‐to‐liquid ratio, pH, and extraction intensity were all reported to affect TPC and antioxidant activity. Nevertheless, the reviewed studies suggest that these variables primarily affect mass transfer and solvent accessibility. Increased extraction time generally improved phenolic recovery up to an optimum point, beyond which gains became marginal and the risk of phenolic degradation increased (Rebollo‐Hernanz et al. 2021; Ramos‐Escudero et al. 2024). Similarly, moderate increases in temperature enhanced extraction efficiency by improving solubility and diffusion rates, although excessive temperatures may promote oxidation and degradation of thermolabile compounds.

Another consistent observation from the articles is that extraction optimization often seems to contribute more to phenolic recovery than the extraction technology itself. Studies employing response surface methodology and other optimization approaches frequently reported substantially higher TPC values than studies using fixed extraction conditions (Ramos‐Escudero et al. 2024; De Barros et al. 2023). This finding suggests that variations in reported TPC values across the literature may reflect differences in process optimization rather than inherent differences in extraction technologies. Consequently, direct comparison among studies remains challenging because solvent systems, extraction conditions, pretreatment strategies, and analytical methods differ considerably. Hence, efficient extraction of CBS polyphenols requires balancing solvent polarity with extraction conditions that promote mass transfer while preserving phenolic stability.

### 4.3 Emerging Extraction Techniques

Various extraction techniques were reported in the reviewed literature for the recovery of polyphenols from CBS, as shown in Table 3. In studies where a comparison was made between the conventional solvent extraction (maceration, Soxhlet) and green extraction techniques (UAE, SWE, MAE, HVED, DES, or PEF), the green techniques were reported to increase the extraction yield, content of polyphenols, as well as the antioxidant activities in CBS, while reducing the extraction time and solvent requirement (Jokić et al. 2018; Ruesgas‐Ramón et al. 2019; Barišic et al. 2020; Mellinas et al. 2020; Jokić et al. 2020; Ramos et al. 2023; Švarc‐Gajić et al. 2023; Benítez‐Correa et al. 2023, 2024). The findings from the included papers are discussed in the following sections, organized by the green extraction technique applied.

To facilitate comparison among studies, TPC values were standardized, where possible, to mg GAE g^−1^ dry weight (DW), the most commonly reported unit for CBS extracts. Following conversion, reported TPC values ranged from approximately 40 to over 260 mg GAE g^−1^ DW depending on extraction technology and processing conditions. UAE generally yielded between 50 and 164 mg GAE g^−1^ DW, whereas MAE produced 17 to 42 mg GAE g^−1^ DW under optimized conditions. Emerging technologies such as PEF, HVED, and subcritical water extraction achieved comparable or higher phenolic recoveries, reaching up to 130 mg GAE g^−1^ DW. Nevertheless, direct comparison remains challenging because several studies reported results as mg GAE mL^−1^ extract, mg GAE L^−1^ extract, percentage recovery, or catechin equivalents, preventing accurate conversion to a common DW basis.

#### 4.3.1 Microwave‐Assisted Extraction

MAE can enhance mass transfer through rapid volumetric heating, reduce extraction time, and decrease solvent consumption compared with conventional extraction methods. Across the reviewed studies, MAE consistently improved the recovery of phenolic compounds and antioxidant activity; however, extraction efficiency was strongly influenced by solvent composition, pH, temperature, and extraction duration. A common trend across the studies is that optimizing extraction conditions can have a greater impact on polyphenol recovery than the microwave treatment itself.

The performance of MAE is strongly influenced by operating parameters, particularly pH and extraction time. Among these, pH has been identified as one of the most influential factors, with alkaline conditions generally promoting greater phenolic recovery and antioxidant activity than neutral or acidic media (Mellinas et al. 2020). This enhancement is primarily attributed to the cleavage of ester and ether linkages between phenolic compounds and cell wall polysaccharides, facilitating the release of bound phenolics (Kumar et al. 2025). Extraction time also plays an important role, as prolonged microwave exposure can increase the release of phenolic compounds until equilibrium is reached (Rahayu et al. 2019). However, extending extraction beyond the optimal duration may increase the likelihood of thermal degradation of thermolabile phenolics and flavonoids, ultimately reducing antioxidant capacity (Pokkanta et al. 2022). These findings highlight that maximizing MAE efficiency requires optimizing multiple processing parameters simultaneously rather than adjusting any single variable.

Solvent selection is a critical determinant of extraction efficiency in MAE. Aqueous organic solvent systems generally outperform water or pure organic solvents because their intermediate polarity enhances the solubilization of structurally diverse phenolic compounds present in CBS (Nsor‐Atindana et al. 2012; Brglez Mojzer et al. 2016; Sukackas et al. 2025). Nevertheless, solvent composition is only one of several factors influencing extraction performance, as process variables such as pH, temperature, microwave power, and extraction time also strongly affect phenolic recovery and extract quality. Consequently, studies that evaluate solvent effects without systematically optimizing these operational parameters may yield only a partial understanding of the factors governing extraction efficiency, thereby limiting direct comparisons with investigations that employ comprehensive process optimization (Mellinas et al. 2020).

When considered collectively, these studies indicate that MAE performance is governed by the interaction of multiple extraction variables rather than any single factor. While extraction time and solvent composition influence phenolic recovery, evidence suggests that pH and temperature exert the strongest effects, as they directly influence cell wall disruption and the release of bound phenolics. Direct comparisons of TPC values remain challenging because studies employed different solvents, extraction conditions, and reporting units, with some reporting results as mg GAE g^−1^ DW and others as mg GAE mL^−1^ extract.

Despite the demonstrated effectiveness of MAE, several research gaps remain. Most studies relied primarily on TPC measurements and antioxidant assays, which provide limited information regarding individual phenolic compounds. Furthermore, the effects of microwave power, phenolic stability during extraction, and scalability for industrial applications remain insufficiently investigated. Future studies should integrate comprehensive phenolic profiling techniques such as HPLC‐MS or LC‐MS/MS with process optimization approaches to better understand how MAE conditions influence both the quantity and composition of recovered polyphenols. Such information is essential for maximizing the valorization potential of CBS as a sustainable source of bioactive compounds.

#### 4.3.2 Hydrothermal Extraction

Among the articles reviewed, two studies (Sánchez, Bernal, Laca et al. 2024; Sánchez, Penin, Laca et al. 2024) employed HTE as a green method for polyphenol extraction, using water as the primary solvent.  HTE employs pressurized hot water to recover bioactive compounds from plant matrices. Among hydrothermal technologies, autohydrolysis is a catalyst‐free process in which the self‐ionization of water and organic acids naturally released from the biomass promote the hydrolysis of structural polysaccharides, thereby enhancing the liberation of bound phenolic compounds (Freitas et al. 2022). The authors exploited the altered dielectric properties of water at elevated temperatures to enhance the solubilization of phenolic compounds from CBS, eliminating or reducing the need for organic solvents. Both studies demonstrate that hydrothermal treatments can effectively recover phenolic compounds from CBS while simultaneously generating extracts with significant antioxidant activity. However, extraction efficiency is highly dependent on processing severity, including temperature, pressure, pH, and treatment duration.

A consistent trend across the studies is that increasing the severity of hydrothermal treatment enhances phenolic extraction (Sánchez, Bernal, Laca et al. 2024; Sánchez, Penin, Laca et al. 2024). The authors attributed this effect to enhanced cell wall disruption and partial degradation of lignin structures at higher temperatures, thereby facilitating the release of phenolic compounds previously bound within the lignocellulosic matrix. This trend is consistent with findings from other lignocellulosic matrices, where elevated temperatures promote cell wall disruption, cleavage of ester linkages, and partial lignin depolymerization, thereby facilitating the release of bound phenolics (Yuan et al. 2021). Similar enhancements in phenolic recovery have been reported for agro‐industrial residues such as grape pomace, coffee grounds, and fruit peels (Mellinas et al. 2020; Agudelo et al. 2021). However, as severity increases, thermal degradation phenomena occur, representing a critical limitation.

The influence of processing conditions is further illustrated by the HTE approach used for functional beverage production (Sánchez, Bernal, Laca et al. 2024; Sánchez, Bernal, Laca et al. 2024). Hydrothermal processing under relatively mild conditions can effectively recover phenolic compounds while preserving their bioactivity, making it a promising green extraction strategy for beverage applications. Furthermore, coupling HTE with fermentation has been shown to enhance the functional properties of the resulting extracts, likely through microbial biotransformation of phenolic compounds and the generation of additional antioxidant metabolites. Similar mechanisms have been reported in fermented cereal, fruit, and tea‐based beverages, where lactic acid bacteria promote the release of bound phenolics and the formation of bioactive compounds with improved antioxidant potential (Yang et al. 2025; de Wolf et al. 2025). These findings highlight the synergistic potential of integrating green extraction technologies with fermentation to develop value‐added functional beverages from CBSs.

HTE efficiency is governed by the balance between maximizing phenolic release and minimizing the formation of thermal degradation products (Sánchez, Bernal, Laca et al. 2024). As processing severity increases, greater disruption of the lignocellulosic matrix can enhance the extraction of phenolic compounds; however, excessive temperatures and prolonged treatment may also promote the formation of undesirable compounds, including hydroxymethylfurfural (HMF), furfural, and organic acids. Consequently, optimizing hydrothermal processing requires careful selection of operating conditions to maximize bioactive compound recovery while preserving extract quality and minimizing degradation product formation.

Another important observation is that HTE achieves phenolic recoveries comparable to those of several solvent‐based extraction methods reported for CBS, while offering advantages in environmental sustainability and solvent safety. The ability of water at elevated temperatures and pressures to alter its physicochemical properties enhances the solubilization of phenolic compounds and reduces reliance on organic solvents. Consequently, HTE aligns well with current trends toward greener and more sustainable biorefinery processes for agro‐industrial by‐products. Overall, the combined evidence from Sánchez, Bernal, Laca et al. 2024; Sánchez, Penin, Laca et al. 2024) demonstrates that HTE is an effective and sustainable strategy for recovering polyphenols from CBS, with extraction severity acting as a key determinant of yield and composition. However, optimal utilization requires balancing extraction efficiency with compound stability and accounting for downstream processes, such as fermentation, to enhance bioactivity.

#### 4.3.3 Ultrasound‐Assisted Extraction

UAE was used in 20 studies in this review. Considerable variability in extraction performance was observed across studies. These differences largely reflect variations in extraction parameters, pretreatment conditions, solvent composition, ultrasound equipment, optimization strategies, and analytical reporting, rather than indicating inconsistencies in the technology itself. The evidence demonstrates that UAE efficiency depends on the interaction between cavitation intensity and mass‐transfer conditions rather than on any single extraction variable.

Across the reviewed studies, aqueous ethanol was consistently identified as the most effective solvent for UAE, with ethanol concentrations around 40%–50% producing higher polyphenol recoveries than either pure water or highly concentrated ethanol. This trend was reported by Grassia et al. (2021), Ramos‐Escudero et al. (2024), and several other UAE studies (Quiceno‐Suárez et al. 2024; Valencia et al. 2024; Llerena et al. 2023; Ramos et al. 2023; Duque et al. 2024; Disca et al. 2024; Avilés et al. 2019), suggesting that solvent polarity plays a more important role than extraction technology alone. Intermediate ethanol concentrations provide sufficient polarity to dissolve hydrophilic phenolics while simultaneously disrupting interactions between polyphenols and the lignocellulosic matrix. Conversely, increasing ethanol concentration beyond approximately 50% reduces solvent polarity and limits the extraction of highly polar flavan‐3‐ols such as catechin and epicatechin, the predominant phenolics in CBS.

Similarly, extraction time exhibits a non‐linear relationship with polyphenol recovery during UAE. Although ultrasound substantially reduces extraction time compared with conventional extraction methods, prolonged sonication does not necessarily improve extraction efficiency (Ramos‐Escudero et al. 2024; Grassia et al. 2021). The rapid cavitation generated during the initial stages of extraction effectively disrupts the cellular matrix of the CBS, accelerating the release of phenolic compounds until equilibrium is approached. Beyond this point, additional sonication generally provides minimal improvement in extraction yield while increasing the risk of localized heating, free‐radical formation, and degradation of thermolabile phenolic compounds. These findings indicate that optimizing extraction time is essential to maximize phenolic recovery while preserving extract quality and improving the energy efficiency of UAE processes.

Ultrasound intensity is another important factor influencing extraction efficiency, although comparisons across studies should be interpreted with caution because different ultrasound systems produce markedly different cavitation conditions. Probe (sonotrode) systems generally deliver ultrasonic energy directly into the extraction medium, producing more intense cavitation and greater disruption of the plant matrix than conventional ultrasonic baths do, thereby enhancing the recovery of phenolic compounds (Ramos‐Escudero et al. 2024; Avilés et al. 2019). Consequently, differences in extraction performance among studies may reflect variations in equipment configuration and energy transfer rather than the intrinsic effectiveness of UAE itself. This highlights a key limitation of the current literature, where extraction efficiencies are frequently compared without standardized reporting of parameters such as energy density, acoustic power, or energy input, making direct comparisons and process reproducibility challenging.

Particle size further demonstrates the synergistic interaction between sample pretreatment and UAE. Reducing the particle size of CBSs increases the available surface area for solvent penetration and shortens diffusion pathways, thereby enhancing mass transfer and facilitating the release of phenolic compounds (Grassia et al. 2021). When combined with ultrasonic cavitation, these physical changes promote more effective disruption of the plant matrix and improve extraction efficiency. Consequently, differences in particle size and milling procedures may contribute substantially to the variability observed among UAE studies and should be considered alongside extraction parameters when comparing process performance.

In addition, studies have adopted multivariate optimization techniques, such as response surface methodology, to evaluate the combined effects of extraction variables rather than assessing individual factors independently (Ramos‐Escudero et al. 2024; Duque et al. 2024). This methodological shift reflects the recognition that interactions among process variables often have a greater influence on extraction performance than any single parameter alone. UAE should be considered an integrated extraction process in which solvent composition, particle size, extraction time, ultrasound intensity, temperature, and solid‐to‐solvent ratio interact to determine phenolic recovery and extract quality. Consequently, systematic optimization of these interdependent variables is essential for maximizing extraction efficiency.

#### 4.3.4 DES Extraction

In this review, two studies (Ruesgas‐Ramón et al. 2020; Benítez‐Correa et al. 2023) used DES to pretreat CBS prior to polyphenol extraction, offering a sustainable alternative to conventional organic solvents. However, the collective evidence indicates that extraction efficiency is governed by the interplay between solvent composition, physicochemical properties, and mass‐transfer characteristics rather than by the use of DES alone. Across the studies, improvements in TPC are consistently associated with optimizing hydrogen‐bond donor strength, water content, and solvent viscosity, demonstrating that DES performance depends on tailoring solvent properties to the characteristics of the CBS matrix rather than on adopting a universal formulation.

Choline chloride‐based DES containing organic acids were reported to outperform polyol‐based DES for extracting phenolic compounds from CBSs. Among the various formulations investigated, choline chloride–lactic acid consistently demonstrated superior extraction performance compared with choline chloride–glycerol, choline chloride–ethylene glycol, and conventional hydro‐organic solvents (Benítez‐Correa et al. 2023; Ruesgas‐Ramón et al. 2020). This enhanced extraction efficiency is attributed not only to the solvent's polarity but also to its capacity to form strong hydrogen‐bond interactions with phenolic compounds, thereby improving their solubilization and release from the lignocellulosic matrix. Collectively, these findings suggest that the molecular interactions between DES components and target phytochemicals are key determinants of extraction performance, highlighting the importance of solvent design in developing efficient and sustainable extraction systems.

The superiority of organic acid‐based DES appears to result from their physicochemical characteristics. Unlike polyol‐based solvents, lactic acid forms stronger hydrogen‐bond networks that disrupt interactions between phenolic compounds and the lignocellulosic matrix while maintaining sufficient polarity to solubilize flavan‐3‐ols (Rente et al. 2021). However, excessive intermolecular interactions also increase solvent viscosity, creating resistance to mass transfer. Consequently, solvent performance reflects a balance between extraction affinity and diffusional limitations. This explains why studies consistently report improvements when DES is diluted with water rather than using undiluted (neat) DES formulations, whose high viscosity limits mass transfer.

Water content has emerged as one of the principal variables controlling the extraction efficiency of DES. Although excessive water addition has traditionally been expected to disrupt the hydrogen‐bond network responsible for DES functionality, studies on CBSs indicate that moderate water incorporation can improve phenolic recovery (Benítez‐Correa et al. 2023). This enhancement may be primarily attributed to reduced solvent viscosity, which facilitates mass transfer and solvent penetration into the lignocellulosic matrix while maintaining sufficient hydrogen‐bond interactions to preserve solvent functionality. These findings suggest that the improved performance of hydrated DES results from an optimal balance between transport properties and solvent–solute interactions rather than changes in solvent chemistry alone.

The importance of mass transfer is further supported by kinetic analyses of DES extraction. Diffusion‐based modeling has shown that polyphenol extraction from CBSs is characterized by an initial rapid release of readily accessible compounds, followed by a slower, diffusion‐controlled stage as phenolics migrate from the particle interior to the solvent (Benítez‐Correa et al. 2023). These mechanistic studies indicate that lowering solvent viscosity enhances internal diffusion and accelerates extraction kinetics, thereby providing a clearer understanding of the transport phenomena that govern DES performance. Such mechanistic approaches represent a significant advancement over studies that compare extraction yields alone, as they help explain why specific DES formulations achieve superior extraction efficiency and provide a stronger basis for process optimization and scale‐up.

The key takeaway from the findings indicates that successful DES extraction of polyphenols from CBSs depends on optimizing the solvent's physicochemical properties rather than indiscriminately selecting a DES formulation. Organic acid‐based DES, particularly choline chloride–lactic acid systems containing moderate amounts of water, consistently provide superior polyphenol recovery by balancing strong solute–solvent interactions with improved mass transfer. From an industrial perspective, DES extraction offers several advantages over conventional organic solvent systems, including reduced toxicity, low volatility, biodegradability, and the ability to tailor the solvent composition to target specific classes of bioactive compounds. Nevertheless, challenges remain before industrial implementation can be achieved. The relatively high viscosity of DES increases the energy requirements during mixing and downstream processing, while solvent recovery, recycling, extract purification, and regulatory acceptance for food applications remain insufficiently investigated. Furthermore, most published studies have been conducted at laboratory scale, and information regarding process economics, continuous extraction systems, and large‐scale solvent regeneration is limited. Future research should focus on standardized extraction protocols, solvent recyclability, techno‐economic assessment, and pilot‐scale validation to facilitate industrial application of DES for sustainable CBS valorization.

#### 4.3.5 PEF Extraction

PEF pretreatment enhances the recovery of phenolic compounds from CBSs compared with conventional extraction methods. This improvement is attributed to electroporation‐induced permeabilization of cell membranes, which facilitates solvent penetration and promotes the release of intracellular bioactive compounds (Barbosa‐Pereira et al. 2018; Benítez‐Correa et al. 2024). However, extraction efficiency is determined not only by PEF treatment but also by its interaction with downstream extraction variables, including solvent composition, extraction time, electric field strength, pulse number, and other processing conditions. Collectively, the findings indicate that the effectiveness of PEF depends on the integrated optimization of pretreatment and extraction parameters, reinforcing its potential as a sustainable technology for improving phenolic recovery from CBSs.

The results from the reviewed studies further indicates that extraction performance depends not only on PEF intensity but also on the properties of the extraction medium. Solvent composition influences both the efficiency of electroporation during pretreatment and the subsequent recovery of phenolic compounds, highlighting the need to optimize solvent characteristics alongside electrical treatment parameters (Barbosa‐Pereira et al. 2018; Benítez‐Correa et al. 2024). In particular, the electrical conductivity of the pretreatment medium plays a critical role in determining the extent of cell membrane permeabilization and the effectiveness of mass transfer. Consequently, the selection of an appropriate pretreatment medium should consider both its electrical properties and its compatibility with the downstream extraction solvent to maximize phenolic recovery. These findings demonstrate that solvent selection in PEF‐assisted extraction extends beyond solvent polarity alone and requires consideration of the electrochemical environment governing electroporation.

Another recurring observation is that increasing PEF intensity does not necessarily produce proportional improvements in extraction efficiency. Instead, an optimal processing appears to exist, beyond which further increases in electric field strength or pulse number provide limited additional benefits and may even reduce the recovery of specific phenolic compounds (Barbosa‐Pereira et al. 2018; Benítez‐Correa et al. 2024). This behavior likely reflects a balance between enhanced cell membrane permeabilization and the potential degradation of sensitive bioactive compounds due to localized heating and electrochemical reactions. Consequently, effective PEF processing requires optimization of treatment intensity to maximize extraction while preserving phenolic stability.

Beyond improving phenolic recovery, PEF‐assisted extraction has also been shown to enhance the extraction of other bioactive constituents, including methylxanthines such as theobromine and caffeine, while increasing the antioxidant potential of the resulting extracts (Benítez‐Correa et al. 2024). Furthermore, when integrated with natural deep eutectic solvent (NADES) extraction, PEF‐generated extracts have demonstrated antimicrobial activity against foodborne pathogens, highlighting the potential of this combined approach to produce multifunctional extracts with applications extending beyond enhanced extraction efficiency. These findings reinforce the value of PEF as a technology that can improve both the yield and functional properties of CBS extracts.

Collectively, PEF is an effective green pretreatment technology for CBS valorization. The enhanced extraction yields reported across studies appear to result primarily from improved cell permeabilization and mass transfer rather than direct effects on phenolic chemistry. However, extraction performance is highly dependent on the interaction among electrical parameters, solvent systems, and extraction conditions. Future studies should therefore focus on standardizing PEF operating parameters and evaluating their effects on individual phenolic compounds to facilitate more robust comparisons and support industrial‐scale implementation.

#### 4.3.6 Supercritical Fluid and Subcritical Water Extraction

Supercritical and subcritical fluid technologies offer environmentally friendly alternatives to conventional solvent extraction and enable the selective recovery of bioactive compounds. Across the reviewed studies, extraction efficiency was governed primarily by the interaction between pressure, temperature, solvent composition, and fluid density, highlighting that process optimization is more important than any individual operating parameter.

Evidence across studies shows that the physicochemical properties of the extraction medium strongly influence both extraction yield and phenolic recovery during SFE. Although supercritical carbon dioxide is an attractive green solvent because of its low toxicity, non‐flammability, and ease of removal, its inherently low polarity limits the extraction of phenolic compounds. The incorporation of polar co‐solvents, particularly ethanol, has consistently been shown to enhance the recovery of phenolics and other polar bioactive constituents by increasing the solvating power of the supercritical fluid (Pico Hernández et al. 2019; Jokić et al. 2018). These findings demonstrate that solvent polarity remains a key determinant of extraction performance, even in advanced fluid‐based extraction systems, and highlight the importance of tailoring solvent composition to the chemical characteristics of the target compounds.

The available evidence also indicates that pressure and temperature exert interdependent effects on SFE performance. Increasing pressure generally enhances solvent density and mass transfer, thereby improving the solubilization of target compounds. In contrast, the influence of temperature is more complex because it simultaneously affects solvent density, diffusivity, solute vapor pressure, and the thermal stability of phenolic compounds (Pico Hernández et al. 2019; Jokić et al. 2018; Švarc‐Gajić et al. 2023). Extraction efficiency therefore depends on balancing these competing effects rather than maximizing pressure or temperature independently. This interaction underscores the need for integrated optimization of SFE operating conditions to maximize phenolic recovery while preserving the integrity of thermolabile bioactive compounds.

Another important trend observed is the differing selectivity of supercritical and subcritical fluid systems. Supercritical CO_2_‐based extraction showed particular effectiveness for recovering lipophilic constituents and specific phenolic fractions, whereas subcritical water extraction (SWE) demonstrated broader capability for extracting polar phenolic compounds due to the reduction in water polarity at elevated temperatures. Jokić et al. (2020) and Švarc‐Gajić et al. (2023) reported that subcritical water extraction yielded extracts with high phenolic content and antioxidant activity, indicating that subcritical water can serve as an effective green solvent for CBS valorization. When combined, these findings suggest that the choice between SFE and SWE should depend on the target compounds and desired extract characteristics rather than on extraction yield alone.

A recurring finding across all studies is that higher extraction yields do not necessarily correspond to higher phenolic concentrations. Several authors reported that conditions that maximize total extract recovery differ from those that maximize phenolic content or antioxidant activity, reflecting the complex composition of CBS and the varying solubility of its constituents. This distinction highlights the importance of evaluating extract quality alongside extraction yield when assessing process performance. Consequently, optimization strategies should focus not only on maximizing recovery but also on enhancing the selective extraction of bioactive compounds. The reviewed literature demonstrates that supercritical and subcritical fluid technologies are effective green extraction approaches for CBS, offering advantages in solvent safety, extract purity, and process sustainability. However, extraction performance is highly dependent on the interaction among pressure, temperature, solvent composition, and extraction time.

#### 4.3.7 OH Extraction

OH was used as a green polyphenol extraction technique with ethanol–water mixtures, with temperature, time, and ethanol concentration optimized via a 2^3^ central composite design (CCD) to maximize TPC and antioxidant activity, as reported by Sánchez et al. (2023). Unlike conventional heating systems that rely on heat transfer from external surfaces, OH generates heat directly within the material by applying an electric current, resulting in rapid and uniform heating. The available evidence suggests that the advantages of OH extend beyond thermal effects, as electrical treatment also induces structural modifications that enhance mass transfer and promote the release of intracellular bioactive compounds.

A consistent trend in the literature is that OH enhances the recovery of phenolic compounds and antioxidant activity from CBSs compared with conventional thermal extraction under comparable processing conditions. The improved extraction performance is attributed not only to rapid and uniform volumetric heating but also to electroporation induced by moderate electric fields, which increases cell membrane permeability and facilitates the diffusion of phenolic compounds from the plant matrix into the extraction medium (Sánchez et al. 2023). These findings suggest that the combined thermal and electrical effects of OH offer advantages over conventional heating by enhancing mass transfer while upholding the principles of green extraction.

The enhanced extraction efficiency achieved by OH appears to be closely linked to structural changes induced in the CBS matrix. Morphological analysis revealed that OH‐treated samples exhibited extensive surface ruptures, pores, and damage to cellular structures, whereas untreated and conventionally heated samples maintained comparatively intact surfaces (Xu et al. 2026). These physical modifications increase solvent accessibility and reduce mass‐transfer limitations, thereby improving the release of bound and intracellular phenolic compounds. Similar mechanisms have been reported for other plant matrices treated with moderate electric fields, suggesting that electroporation is a fundamental contributor to the improved extraction performance observed with OH.

Another important observation is that increased phenolic recovery was accompanied by enhanced biological functionality. Extracts obtained under optimized OH conditions demonstrated strong antioxidant activity and reactive oxygen species (ROS)‐scavenging capacity while maintaining low cytotoxicity toward non‐tumoral cell lines. This finding suggests that OH not only improves extraction efficiency but also preserves the bioactivity of the recovered compounds. The technology may offer advantages over more severe thermal treatments that can compromise phenolic stability through oxidation or degradation reactions.

The available evidence further highlights that the effectiveness of OH depends on the integrated optimization of processing conditions rather than the application of electrical treatment alone. Extraction performance is governed by the interaction between solvent composition, temperature, treatment time, and electrical parameters, indicating that these variables should be optimized simultaneously to maximize phenolic recovery and antioxidant activity (Sánchez et al. 2023). This pattern is consistent with other green extraction technologies for CBSs, where process variables and solvent characteristics often exert a greater influence on extraction efficiency than the extraction technology itself. The combination of enhanced cell permeabilization, improved mass transfer, efficient phenolic recovery, and the preservation of antioxidant functionality highlights OH's potential to produce food‐grade and nutraceutical extracts. However, despite these promising outcomes, the current evidence remains limited.

#### 4.3.8 HVED Extraction

Among the articles included in this systematic review, two studies used high‐voltage electrical discharge.  Jokić et al. (2019) used HVED as an extraction technique, while Barišic et al. (2020) used HVED in water as a treatment step followed by solvent extraction. HVED has great potential as a non‐thermal green extraction technology for recovering bioactive compounds because it generates electrical discharges in liquid media that create shock waves, cavitation, and localized cell disruption, thereby enhancing mass transfer while avoiding the high temperatures that can degrade thermolabile phenolic compounds. Across the reviewed studies, the principal advantage of HVED was not simply higher extraction yields but its ability to improve the selective release of intracellular bioactive compounds using water‐based extraction systems and relatively low energy inputs.

The studies by Jokić et al. (2019) and Barišić et al. (2020) consistently demonstrate that HVED performance depends more strongly on process optimization than on the technology itself. Both studies identified extraction frequency, treatment time, and solid‐to‐solvent ratio as the dominant factors influencing phenolic recovery, indicating that HVED is highly sensitive to operational conditions. Although both investigations used water‐based systems, they pursued different objectives. Jokić et al. (2019) focused on maximizing extraction efficiency from ground CBS, whereas Barišić et al. (2020) evaluated the retention of phenolic compounds and methylxanthines following HVED treatment of cocoa shell suspensions. Together, these studies suggest that HVED can either enhance extraction or preserve bioactive compounds depending on how the process is configured.

Moderate high‐voltage electrical discharge processing conditions generally provide more favorable outcomes than prolonged or excessively intense treatments. Appropriate optimization of electrical parameters enhances cell disruption and promotes the release of intracellular phenolic compounds and other bioactive constituents, whereas excessive treatment intensity may increase oxidation or degradation of thermolabile phenolics, thereby reducing the overall effectiveness of the extraction process (Jokić et al. 2019; Barišić et al. 2020). These findings indicate that maximizing HVED performance requires balancing efficient tissue disruption with the preservation of bioactive compound stability rather than simply increasing treatment intensity.

The available evidence also demonstrates that the perceived effectiveness of HVED depends on the intended application of the process. Some studies have focused on maximizing the recovery of bioactive compounds into extracts, whereas others have evaluated the preservation of phenolic compounds within treated CBSs for subsequent food applications (Jokić et al. 2019; Barišić et al. 2020). Consequently, differences in reported phenolic contents should be interpreted in the context of the extraction objective, analytical endpoint, and experimental design rather than as direct measures of extraction efficiency alone. This methodological diversity contributes to the variability reported across the literature and highlights the need for standardized processing protocols and reporting practices to enable more meaningful comparisons of HVED performance.

From a broader perspective, HVED occupies an intermediate position among green extraction technologies. Compared with conventional extraction, it offers clear advantages by reducing thermal damage, improving mass transfer, and enabling the use of environmentally friendly solvents such as water. However, unlike more established technologies such as UAE or subcritical water extraction, the evidence base for HVED remains limited to a relatively small number of studies with varying experimental designs. Furthermore, the absence of standardized operating conditions and limited information on process scalability make direct comparisons with other green extraction techniques difficult.

#### 4.3.9 Combined Extraction Techniques

Hybrid extraction technologies have attracted considerable attention because they integrate complementary physical mechanisms that can intensify mass transfer while reducing extraction time and solvent consumption (Hndeya et al. 2026). In principle, combining UAE with MAE, PLE, or green solvents such as DESs should improve cell‐wall disruption and facilitate the release of intracellular polyphenols. However, the studies included in this review demonstrate that combining extraction technologies does not consistently produce higher TPC, indicating that synergistic effects are highly dependent on extraction conditions and the physicochemical characteristics of the CBSs matrix.

Comparative studies evaluating multiple extraction techniques indicate that combining green extraction technologies does not necessarily produce greater phenolic recovery than optimized individual methods (Disca et al. 2024). Instead, improvements in antioxidant activity may arise from the extraction of other bioactive constituents, including high‐molecular‐weight melanoidins and Maillard reaction products formed during cocoa processing. This finding highlights that TPC and antioxidant capacity are not always directly correlated, particularly in roasted CBSs, where non‐phenolic antioxidants can make substantial contributions to radical‐scavenging activity. Consequently, assessing extraction performance solely on the basis of TPC may underestimate the functional value of extracts, underscoring the importance of using complementary chemical and antioxidant analyses when evaluating extraction technologies.

The absence of synergistic improvements in TPC can be explained by the distinct mechanisms governing microwave and ultrasonic extraction. Microwave irradiation rapidly heats intracellular moisture through dipole rotation and ionic conduction, generating internal pressure that disrupts plant tissues and accelerates solvent penetration (Reina et al. 2026). In contrast, ultrasound enhances extraction primarily through acoustic cavitation, which produces microjets and shock waves that erode cell walls and reduce the diffusion boundary layer around plant particles (Hoo et al. 2022). When both technologies are applied simultaneously, the benefits are not necessarily additive. Once sufficient structural disruption has occurred, polyphenol release becomes limited by the finite concentration of extractable compounds rather than by mass‐transfer resistance. Consequently, additional physical energy may increase the extraction of non‐phenolic constituents instead of increasing phenolic recovery.

In addition, excessive energy input may adversely affect extraction efficiency. Localized heating generated by microwave irradiation, together with cavitation‐induced shear forces during ultrasound treatment, can promote oxidation, hydrolysis, polymerization, or structural transformation of thermolabile phenolic compounds, particularly flavan‐3‐ols such as catechin and epicatechin (Nayak et al. 2024). Simultaneously, elevated temperatures accelerate the co‐extraction of proteins, polysaccharides, lignin fragments, and melanoidins, thereby increasing extract complexity without a proportional increase in TPC. As reported by Disca et al. (2024), combined UAE‐MAE treatments extracted greater quantities of brown melanoidin‐rich fractions than individual extraction methods, supporting the hypothesis that hybrid processes increasingly recover high‐molecular‐weight compounds that contribute to antioxidant activity but are not quantified as phenolics by chromatographic analysis.

The structural characteristics of the CBSs also play a critical role in determining the effectiveness of hybrid extraction systems. The lignocellulosic matrix, composed primarily of cellulose, hemicellulose, and lignin, retains a proportion of phenolic compounds through interactions with structural polymers, limiting their accessibility to extraction solvents (Quiceno‐Suárez et al. 2024). Although hybrid extraction approaches can enhance matrix disruption and facilitate the release of bound phenolics, their effectiveness remains strongly influenced by the raw materials’ intrinsic properties. Variations in cocoa genotype, fiber composition, lignin content, roasting conditions, particle size, and residual lipid content affect solvent penetration and mass transfer, thereby influencing the fraction of phenolic compounds that can be recovered. These findings indicate that, beyond a certain point, extraction efficiency is constrained by matrix composition rather than extraction intensity alone, emphasizing the importance of considering raw material characteristics alongside process optimization when developing hybrid extraction strategies.

These observations help explain the inconsistent performance of hybrid extraction technologies reported across the literature. The effectiveness of combined techniques depends on achieving an appropriate balance between structural disruption and preservation of phenolic stability. Moderate extraction intensities enhance solvent penetration and diffusion, whereas excessive temperatures, prolonged exposure, or unnecessary combinations of physical treatments may increase degradation reactions or favor the extraction of non‐phenolic antioxidants. Therefore, the superiority of hybrid extraction cannot be assumed solely from the combination of technologies. Instead, extraction performance depends on careful optimization of operating variables—including temperature, extraction time, ultrasound power, microwave energy, solvent composition, and solid‐to‐solvent ratio—to maximize polyphenol recovery while minimizing degradation and non‐selective co‐extraction.

### 4.4 Comparative Assessment of Extraction Technologies

While individual studies provide valuable insights into the performance of specific green extraction technologies, direct comparisons are often complicated by differences in extraction conditions, solvents, analytical methods, and optimization strategies. Therefore, a broader comparative evaluation is necessary to identify the relative strengths, limitations, and industrial potential of each approach. Table 5 synthesizes evidence from the reviewed studies by comparing the principal green extraction technologies across extraction efficiency, solvent sustainability, processing time, energy demand, scalability, industrial readiness, and key limitations. This comparative assessment provides a practical framework for evaluating the suitability of each technology for the sustainable recovery of bioactive compounds from CBSs and highlights areas requiring further technological development and process optimization.

**TABLE 5:** Comparative evaluation of green extraction technologies for the valorization of cocoa bean shells.

Extraction technique	Extraction efficiency	Solvent sustainability	Processing time	Energy demand	Scalability	Industrial readiness	Principal limitation	Overall assessment	References
Conventional solvent extraction (CSE)	Moderate	Moderate	Long	Moderate	Excellent	Established	High solvent consumption	Baseline technology	Jokić et al. (2018), Ruesgas‐Ramón et al. (2019), Barišić et al. (2020)
Ultrasound‐assisted extraction (UAE)	High	High	Short	Low–moderate	High	Commercially available	Limited penetration in dense matrices	Excellent balance between efficiency and sustainability	Ramos et al. (2023), Valencia et al. (2024), Llerena et al. (2023), Quiceno‐Suarez et al. (2025), Gil‐Ramírez et al. (2024), Grassia et al. (2021), Avilés et al. (2019), Grillo et al. (2019), Duque et al. (2024)
Microwave‐assisted extraction (MAE)	High	High	Very short	Moderate	Moderate–high	Commercial	Equipment cost and scale‐up challenges	Highly promising for rapid extraction	Mellinas et al. (2020), Nsor‐Atindana et al. (2012), Rahayu et al. (2019)
Subcritical water extraction (SWE)	Very high	Excellent	Moderate	High	Moderate	Emerging	High‐pressure equipment required	Highly efficient solvent‐free technology	Jokić et al. (2018), Pico Hernández et al. (2019), Švarc‐Gajić et al. (2023)
High‐voltage electric discharge (HVED)	Moderate–High	Excellent	Very short	Moderate	Low–moderate	Emerging	Lack of standardized operating conditions and limited industrial validation	Promising but requires further optimization	Jokić et al. (2019), Barišić et al. (2020)
Pulsed electric field (PEF)	High (mainly used as pretreatment)	Excellent	Very short	Low	High	Commercial for food processing	Usually requires a combination with another extraction method	Effective pretreatment technology	Barbosa‐Pereira et al. (2018), Benítez‐Correa et al. (2024)
Deep eutectic solvent (DES) extraction	High	Very high	Moderate	Low	Moderate	Emerging	Solvent recovery and regulatory acceptance	Sustainable alternative with strong future potential	Benítez‐Correa et al. (2023), Ruesgas‐Ramón et al. (2020)
Ohmic heating (OH)	High	High	Short	Moderate	Moderate	Emerging	Limited industrial applications for CBS extraction	Efficient and energy‐saving technology	Sánchez et al. (2023)
Hydrothermal extraction (autohydrolysis)	Very high	Excellent	Moderate	High	Moderate	Emerging	High‐pressure equipment required	Highly efficient solvent‐free technology	Sánchez, Bernal, Laca et al. 2024; Sánchez, Penin, Laca et al. 2024

### 4.5 Presence of a Food Matrix

The available evidence in the reviewed papers demonstrates that incorporating CBS into food matrices reduces the extractability and measured concentration of polyphenols compared with CBS analyzed as a standalone raw material (Papillo et al. 2019; Grassia et al. 2021; Jokić et al. 2020; I. D. Soares, Cirilo, et al. 2023; Valencia et al. 2024). Although the extent of this reduction varies among studies, a common finding is that polyphenol recovery is influenced not only by the intrinsic composition of CBS but also by physicochemical interactions between phenolic compounds and the surrounding food matrix. These interactions become increasingly important in complex food systems, including bakery and dairy products, where proteins, dietary fibers, starch, and cell‐wall polysaccharides can reduce the accessibility of phenolic compounds during solvent extraction (Ameer et al. 2017; I. D. Soares, Cirilo, et al. 2023; Rojo‐Poveda et al. 2020; Younes et al. 2023).

Across the reviewed studies, lower TPC values were consistently reported for CBS‐enriched foods than for equivalent quantities of CBS extracted before incorporation into food formulations (De Barros et al. 2020; I. D. Soares, Cirilo, et al. 2023). Despite these reductions, increasing the proportion of CBS added to food products generally resulted in proportional increases in TPC and antioxidant capacity, demonstrating that CBS enrichment remains an effective strategy for enhancing the functional properties of foods (Rojo‐Poveda et al. 2020; De Barros et al. 2020). Collectively, these findings indicate that the food matrix primarily limits the extractability of phenolic compounds rather than eliminating their nutritional or functional potential.

The mechanisms underlying these observations appear to be multifactorial. Phenolic compounds readily interact with proteins, dietary fibers, and polysaccharides through hydrogen bonding, hydrophobic interactions, and other non‐covalent associations, thereby reducing their solubility and diffusion into extraction solvents (Rojo‐Poveda et al. 2020; I. D. Soares, Cirilo, et al. 2023; Younes et al. 2023). Furthermore, the manufacture of CBS‐enriched foods commonly involves thermal processing, such as baking, which can promote oxidation, polymerization, or degradation of thermolabile phenolics. Consequently, the lower phenolic concentrations reported in finished food products are likely to reflect the combined effects of matrix binding and processing‐induced transformations rather than reduced extraction efficiency alone. This distinction is particularly important when comparing studies because differences in formulation, processing conditions, and analytical procedures can substantially influence the reported recovery of phenolic compounds.

Comparison of the available studies further suggests that matrix composition exerts a greater influence on polyphenol recovery than the specific food product itself. Food systems with higher concentrations of proteins, starch, or dietary fiber offer more sites for phenolic binding, thereby limiting solvent accessibility during extraction (I. D. Soares, Cirilo, et al. 2023; Rojo‐Poveda et al. 2020). In addition, variability in roasting conditions, particle size, moisture content, extraction solvents, and analytical methods contributes to the wide range of TPC values reported in the literature (De Barros et al. 2020; I. D. Soares, Cirilo, et al. 2023). These methodological differences hinder direct comparison between studies and highlight the need for standardized extraction protocols and harmonized reporting methods for CBS‐enriched foods.

From the perspective of green extraction technologies, these findings suggest that conventional solvent extraction may underestimate the recoverable polyphenol fraction in complex food matrices. Technologies that enhance matrix disruption, including UAE, MAE, PEF, and HVED, may improve the release of phenolic compounds that remain partially bound within food structures by increasing solvent penetration and mass transfer (Jokić et al. 2019; Barišić et al. 2020; Mellinas et al. 2020; Disca et al. 2024; Cañas et al. 2024). Future research should therefore focus not only on maximizing extraction efficiency but also on understanding matrix–polyphenol interactions and developing extraction protocols specifically optimized for complex food systems. Such approaches will facilitate more accurate quantification of CBS bioactive compounds and support the development of functional foods with improved nutritional value and industrial applicability.

### 4.6 Food Processing Techniques

Food‐processing operations substantially influence the stability, extractability, and functional properties of CBS polyphenols. Although different processing technologies, including baking, extrusion, roasting, and other thermal treatments, vary in their operating conditions, they exhibit common effects on polyphenol recovery. Across the studies, processing intensity rather than the specific technology emerged as the principal factor determining whether polyphenol concentrations decrease, remain stable, or become more extractable. This observation highlights that the preservation of CBS bioactive compounds depends on achieving an appropriate balance between structural disruption and thermal degradation rather than simply minimizing or maximizing processing severity (Pismag et al. 2024; Călinoiu and Vodnar 2020).

An observation across the literature is that high temperatures, prolonged processing times, oxygen exposure, and mechanical shear reduce the concentration of thermolabile phenolic compounds by promoting oxidation, polymerization, and structural degradation (Pismag et al. 2024; Latos‐Brozio and Masek 2020). These effects are particularly evident during extrusion and baking, where thermal and mechanical stresses occur simultaneously. For example, extrusion of cocoa husk‐enriched snack products resulted in slightly lower total polyphenol content than the corresponding non‐extruded formulations, despite identical cocoa husk incorporation levels, suggesting that processing conditions, rather than ingredient concentration, were responsible for the observed reductions (Jozinović et al. 2018). Similarly, studies evaluating CBS‐enriched biscuits, bread, cookies, and sprout‐based products consistently reported reductions in TPC and antioxidant activity following baking, reflecting the susceptibility of catechins, flavonoids, and other thermolabile compounds to prolonged heating (Rojo‐Poveda et al. 2021; Blanch and Ruiz del Castillo 2021; Alfeo et al. 2020; Jabłońska et al. 2024).

Despite this, the reviewed studies also demonstrate that thermal processing is not universally detrimental to polyphenol recovery. Moderate thermal treatments can disrupt cell‐wall structures and weaken interactions between phenolic compounds and lignocellulosic components, thereby increasing the release of previously bound polyphenols and improving their extractability (Călinoiu and Vodnar 2020). Consequently, the final polyphenol concentration measured after processing reflects two competing phenomena: degradation of heat‐sensitive compounds and enhanced liberation of matrix‐bound phenolics. Therefore, whether net polyphenol recovery increases or decreases depends on the balance between these opposing mechanisms, which helps explain the variability observed across different food‐processing studies.

Comparison of the available evidence further suggests that differences among studies are influenced not only by processing technology but also by product formulation and processing conditions. Factors such as processing temperature, residence time, moisture content, particle size, oxygen availability, and the composition of the surrounding food matrix all influence the stability and extractability of CBS polyphenols (Pismag et al. 2024; Rojo‐Poveda et al. 2021). In addition, interactions between phenolic compounds and proteins, starch, dietary fiber, and cell‐wall polysaccharides may promote the formation of insoluble complexes that reduce solvent accessibility, thereby lowering the measured phenolic content after processing, even when degradation is limited (Afoakwa 2014; Younes et al. 2023). These methodological and compositional differences partly explain the considerable variability reported across studies and highlight the importance of standardized processing and analytical protocols when evaluating CBS‐enriched foods.

From an industrial perspective, the collective evidence suggests that preserving the functional value of CBS depends on optimization rather than avoidance of food processing. Processing conditions should be designed to minimize unnecessary thermal exposure while maintaining product safety, quality, and consumer acceptability. Furthermore, integrating optimized food‐processing operations with green extraction technologies, such as UAE, MAE, PLE, PEF, or HVED, offers a promising strategy to improve the recovery of matrix‐bound polyphenols either before incorporation into foods or during downstream processing (Disca et al. 2024; Cañas et al. 2024).

## 5 Gaps in Literature and Study Limitations

Despite the growing interest in green extraction technologies for recovering polyphenols from CBSs, several important knowledge gaps remain, limiting direct comparisons among studies and hindering the translation of laboratory findings into industrial applications. One of the most significant challenges is the considerable methodological heterogeneity across the available literature. Differences in cocoa genotype, geographical origin, fermentation and roasting conditions, particle size, moisture content, extraction solvents, processing parameters, and analytical methods contribute substantially to the variability in reported extraction efficiencies and polyphenol contents. Furthermore, inconsistencies in reporting units, including mg GAE g^−1^ dry weight, mg GAE mL^−1^ extract, g L^−1^, and percentage recovery, complicate quantitative comparisons and limit the feasibility of meta‐analytical approaches.

Another important limitation of the current evidence is the predominance of laboratory‐scale investigations. Although UAE, MAE, subcritical water extraction, DESs, PEF, and HVED technologies have demonstrated promising extraction efficiencies, relatively few studies have evaluated pilot‐scale or industrial‐scale performance. Consequently, important practical considerations, including solvent recovery, process integration, equipment costs, energy consumption, operational stability, and techno‐economic feasibility, remain insufficiently explored. Likewise, comprehensive life‐cycle assessments and environmental impact analyses comparing green extraction technologies are scarce, despite their importance for validating sustainability claims.

The review also identified substantial variability in the analytical characterization of recovered polyphenols. Many studies relied primarily on TPC and antioxidant assays as indicators of extraction performance, whereas relatively few employed advanced analytical techniques such as LC‐MS/MS, UHPLC‐MS, or metabolomic profiling to characterize individual phenolic compounds. This limited understanding of how different extraction technologies influence the qualitative composition, stability, bioaccessibility, and biological activity of recovered phytochemicals. Future research should therefore place greater emphasis on comprehensive compound profiling alongside conventional antioxidant measurements.

Although combinations of emerging extraction technologies have attracted increasing attention, evidence regarding process integration remains limited. Few studies have systematically evaluated hybrid extraction strategies or investigated synergistic interactions between pretreatment methods and green extraction technologies under standardized experimental conditions. Similarly, limited attention has been given to optimizing extraction processes using multi‐objective approaches that simultaneously consider extraction yield, phenolic composition, energy efficiency, environmental sustainability, and economic viability.

Several limitations of the present review should also be acknowledged. The available evidence was characterized by substantial methodological diversity, which precluded quantitative meta‐analysis and limited comparisons among extraction technologies. The review relied on published studies, introducing the possibility of publication bias because studies reporting favorable extraction outcomes are more likely to be published than those with negative or inconclusive findings. In addition, although an adapted risk‐of‐bias framework was developed to systematically evaluate methodological quality, no universally validated assessment tool currently exists for laboratory‐based extraction studies. Consequently, the methodological appraisal should be interpreted as a structured evaluation of study quality rather than a definitive measure of bias.

Future research should prioritize developing standardized reporting guidelines for green extraction studies, including harmonized experimental protocols, consistent reporting units, and minimum methodological reporting requirements. Greater emphasis should also be placed on pilot‐scale validation, solvent recyclability, life‐cycle assessment, techno‐economic analysis, regulatory considerations, integration of advanced analytical techniques to facilitate industrial implementation, downstream processing technologies, such as purification, encapsulation, stabilization, and controlled‐release systems, as well as techniques such as spray drying, freeze‐drying, nanoencapsulation, and liposomal delivery to enhance the stability and bioavailability of CBS polyphenols for incorporation into functional foods and nutraceuticals. Shelf‐life studies and formulation compatibility assessments are also needed to support the commercialization of CBS‐enriched products. Addressing these challenges will improve reproducibility, strengthen comparisons among extraction technologies, and accelerate the sustainable valorization of CBSs as a source of high‐value polyphenols within circular bioeconomy systems.

## 6 Regulatory and Safety Considerations for CBSs‐Derived Ingredients

Although CBSs have considerable potential as a sustainable source of dietary fiber, polyphenols, and methylxanthines for functional food development (Rojo‐Poveda et al. 2020; Sánchez et al. 2025), their industrial application depends not only on extraction efficiency but also on compliance with food safety and regulatory requirements. As an agricultural by‐product, CBS may contain chemical, biological, and physical contaminants originating from cultivation, fermentation, drying, storage, and cocoa processing (Barišić et al. 2020). Consequently, comprehensive safety evaluation is essential before CBS‐derived ingredients can be incorporated into commercial food products.

The principal safety concerns associated with CBS include contamination with heavy metals, particularly cadmium and lead; pesticide residues; mycotoxins such as ochratoxin A and aflatoxins; and microbial contaminants introduced during postharvest handling and storage (Bereda 2026; Subroto et al. 2023; Barišić et al. 2020). The concentration of these contaminants varies according to geographical origin, agricultural practices, processing conditions, and storage environments. Appropriate raw material selection and the implementation of Good Agricultural Practices (GAP), Good Manufacturing Practices (GMP), and Hazard Analysis and Critical Control Point (HACCP) systems are therefore essential to ensure product safety and consistency (FAO and WHO 2023; de Oliveira et al. 2016).

Food processing and green extraction technologies can contribute to improved safety by reducing microbial loads and minimizing the use of potentially hazardous organic solvents (Islam et al. 2022; Nawaz et al. 2026). Water‐based extraction methods, UAE, MAE, subcritical water extraction, and other environmentally friendly techniques are particularly attractive because they reduce solvent residues while preserving thermolabile bioactive compounds (Jokić et al. 2018; Ruesgas‐Ramón et al. 2019; Mellinas et al. 2020; Jokić et al. 2020; Ramos et al. 2023; Švarc‐Gajić et al. 2023; Benítez‐Correa et al. 2023; Quiceno‐Suárez et al. 2024). Nevertheless, extraction processes should be validated to demonstrate that they do not concentrate undesirable contaminants alongside the target phytochemicals.

Regulatory approval remains another important consideration for industrial utilization. Requirements vary across jurisdictions, but manufacturers are generally expected to demonstrate the safety, composition, intended use, and quality of CBS‐derived ingredients prior to commercialization. According to the European Food Safety Authority, novel food ingredients may require a safety assessment under the European Union Novel Food Regulation (EU) 2015/2283 if they have not been consumed to a significant extent before May 1997 (European Parliament and Council of the European Union 1997). Likewise, food ingredients marketed in the United States must comply with applicable US Food and Drug Administration requirements, including demonstrating safety through Generally Recognized as Safe (GRAS) status or other relevant regulatory pathways, where appropriate (Pomeranz et al. 2024). Compliance with contaminant limits established by national and international authorities is also necessary to ensure consumer protection.

Another important consideration is the standardization of CBS‐derived ingredients. Variability in cocoa variety, geographical origin, roasting conditions, particle size, and extraction protocols contributes to significant differences in polyphenol composition and antioxidant activity reported across studies (I. D. Soares, Cirilo et al. 2023; Rojo‐Poveda et al. 2020; Jokić et al. 2020). Establishing standardized quality specifications for bioactive composition, contaminant limits, and processing conditions would facilitate regulatory approval, improve reproducibility, and enhance consumer confidence in CBS‐based functional ingredients.

Finally, the successful commercialization of CBS‐derived ingredients will depend on integrating sustainable extraction technologies with robust quality assurance, contaminant monitoring, standardized manufacturing practices, and compliance with international food safety regulations. Researchers should therefore integrate technological optimization with toxicological assessment, regulatory evaluation, and life‐cycle analysis to support the safe and sustainable use of CBS in the food industry.

## 7 Conclusion

This systematic review provides a comprehensive synthesis of current evidence on green extraction technologies for recovering polyphenols from CBSs, highlighting their potential to transform this abundant agro‐industrial by‐product into a valuable source of bioactive compounds for food, nutraceutical, pharmaceutical, and cosmetic applications. The findings demonstrate that polyphenol recovery is governed not by extraction technology alone but by the complex interactions among raw material characteristics, pretreatment conditions, solvent selection, and process optimization. Variations in cocoa genotype, fermentation, roasting, particle size, solvent composition, extraction parameters, and analytical methodologies contribute substantially to the wide range of extraction efficiencies reported across studies.

Among the reviewed technologies, UAE emerged as the most extensively investigated and currently offers the most balanced combination of extraction efficiency, reduced solvent consumption, short processing times, operational simplicity, and potential for industrial implementation. MAE, subcritical water extraction (SWE), HTE, PEF, HVED, and DES‐based extraction also demonstrated considerable potential to improve polyphenol recovery while reducing the environmental burden associated with conventional solvent extraction. However, no single technology proved universally superior under all experimental conditions, emphasizing that extraction performance depends primarily on appropriate process optimization rather than on the extraction technique itself.

The review also highlights several important challenges that currently limit direct comparison among studies. Considerable methodological heterogeneity exists in terms of raw material preparation, extraction conditions, solvent systems, analytical methods, and reporting units. Furthermore, many studies remain confined to laboratory‐scale investigations, with relatively few evaluating process scalability, solvent recovery, techno‐economic feasibility, life‐cycle environmental impacts, or regulatory considerations necessary for commercial implementation. The absence of standardized reporting protocols further reduces reproducibility and complicates quantitative comparison of extraction efficiencies across technologies.

Overall, the available evidence strongly supports the valorization of CBSs as a sustainable source of high‐value polyphenols within a circular bioeconomy framework. Continued advances in green extraction technologies, combined with standardized methodologies and industrial‐scale validation, will play a critical role in enabling the efficient, environmentally responsible, and economically viable utilization of this underexploited by‐product. Such developments have the potential not only to enhance the sustainability of cocoa processing but also to contribute to the growing demand for naturally derived functional ingredients in next‐generation food and health products.

## Author Contributions

**Joy Chinenye Mba**: conceptualization, methodology, validation, visualization, Writing – review and editing, data curation, Writing – original draft, investigation, software, formal analysis, project administration. **Leda Atílio Pita**: methodology, validation, visualization, writing – review and editing, writing – original draft, software, formal analysis, data curation. **Helen Obioma Agu**: writing – review and editing, visualization, validation, software, data curation. **Chioke Amaefuna Okolo**: writing – review and editing, visualization, formal analysis, data curation. **Carmen Sílvia Fávaro–trindade**: conceptualization, funding acquisition, writing – review and editing, visualization, methodology, validation, software, supervision, data curation, formal analysis, resources, project administration.

## Conflicts of Interest

The authors declare no conflict of interest.
